# Integrative phenotyping analyses reveal the relevance of the phyB-PIF4 pathway in *Arabidopsis thaliana* reproductive organs at high ambient temperature

**DOI:** 10.1186/s12870-024-05394-w

**Published:** 2024-07-29

**Authors:** Shekoufeh Ebrahimi Naghani, Ján Šmeringai, Barbora Pleskačová, Tereza Dobisová, Klára Panzarová, Markéta Pernisová, Hélène S. Robert

**Affiliations:** 1 Hormonal Crosstalk in Plant Development, Mendel Center for Plant Genomics and Proteomics, CEITEC MU—Central European Institute of Technology, Masaryk University, Brno, 625 00 Czech Republic; 2 Laboratory of Functional Genomics and Proteomics, National Centre for Biomolecular Research, Faculty of Science, Masaryk University, Brno, 625 00 Czech Republic; 3 Mendel Center for Plant Genomics and Proteomics, CEITEC MU—Central European Institute of Technology, Masaryk University, Brno, 625 00 Czech Republic; 4 PSI - Photon Systems Instruments, Drasov, 66424 Czech Republic; 5 Labdeers s.r.o, Boskovice, 68001 Czech Republic

**Keywords:** Arabidopsis, Automatic phenotyping, PIF4, Pistils, phyB, Pollen tube guidance, Seeds, Thermomorphogenesis

## Abstract

**Background:**

The increasing ambient temperature significantly impacts plant growth, development, and reproduction. Uncovering the temperature-regulating mechanisms in plants is of high importance, for increasing our fundamental understanding of plant thermomorphogenesis, for its potential in applied science, and for aiding plant breeders in improving plant thermoresilience. Thermomorphogenesis, the developmental response to warm temperatures, has been primarily studied in seedlings and in the regulation of flowering time. PHYTOCHROME B and PHYTOCHROME-INTERACTING FACTORs (PIFs), particularly PIF4, are key components of this response. However, the thermoresponse of other adult vegetative tissues and reproductive structures has not been systematically evaluated, especially concerning the involvement of phyB and PIFs.

**Results:**

We screened the temperature responses of the wild type and several phyB-PIF4 pathway *Arabidopsis* mutant lines in combined and integrative phenotyping platforms for root growth in soil, shoot, inflorescence, and seed. Our findings demonstrate that phyB-PIF4 is generally involved in the relay of temperature signals throughout plant development, including the reproductive stage. Furthermore, we identified correlative responses to high ambient temperature between shoot and root tissues. This integrative and automated phenotyping was complemented by monitoring the changes in transcript levels in reproductive organs. Transcriptomic profiling of the pistils from plants grown under high ambient temperature identified key elements that may provide insight into the molecular mechanisms behind temperature-induced reduced fertilization rate. These include a downregulation of auxin metabolism, upregulation of genes involved auxin signalling, *miRNA156* and *miRNA160* pathways, and pollen tube attractants.

**Conclusions:**

Our findings demonstrate that phyB-PIF4 involvement in the interpretation of temperature signals is pervasive throughout plant development, including processes directly linked to reproduction.

**Supplementary Information:**

The online version contains supplementary material available at 10.1186/s12870-024-05394-w.

## Background

Abiotic stresses affect plant growth through physiological, morphological, biochemical, and molecular changes. Among these stresses, warm ambient temperatures affect plant life differently at different growth stages [1, 2]. With the Intergovernmental Panel on Climate Change (IPCC) predicting a global temperature increase of 1.8 °C to 4 °C by 2100 [3], understanding the physiological responses of plants to warmth and the molecular mechanisms involved is crucial for improving high temperature tolerance.

Temperatures above the critical threshold temperature (about 30 °C for most temperate crops) are considered heat stress, which involves severe impairment of cellular functions (membrane fluidity, oxidative stress, protein folding) and may induce cellular death [4]. The response of plants to heat stress is primarily regulated by HEAT SHOCK FACTOR (HSF) transcription factors which orchestrate the expression of downstream regulators [5]. High ambient temperatures (hAT) below this critical threshold temperature induce responses in *Arabidopsis thaliana* resulting in morphological changes collectively referred to as thermomorphogenesis, such as stem elongation, hyponastic leaves, reduced biomass, and accelerated flowering, which help plants dissipate heat and move organs to cooler environments [6, 7]. Thermomorphogenesis is an adaptive mechanism involving transcriptional changes, hormonal reactions, and developmental modifications. The bHLH PHYTOCHROME-INTERACTING FACTOR (PIF) transcription factors, particularly PIF4, play a crucial role in thermomorphogenesis and act as a central hub coordinating signaling pathways and facilitating the plant’s adjustment to environmental conditions [8, 9]. While thermomorphogenesis and heat shock response are distinct thermal responses to a different temperature range, crosstalk between these two processes has been identified with the finding that HSFA1 proteins are required for PIF4-mediated thermomorphogenesis in hAT [10].

The photoreceptor phytochrome B (phyB) serves as a thermosensor [11–13]. Synthesized in its inactive form (phyB-Pr) in the cytoplasm, it converts into an active form (phyB-Pfr) upon absorption of red light and translocates to the nucleus, where it interacts with various transcription factors, including PIFs, to repress gene expression [14, 15]. phyB-Pfr regulates PIF4 abundance and activity in the nucleus by direct interaction and phosphorylation leading to PIF4 proteasomal degradation [16, 17]. Also, phyB-Pfr associates with the PIF4 G-boxes binding site at promoters of temperature-responsive genes, repressing PIF4 transcriptional activity [18]. hAT promotes the thermal reversion of phyB from Pfr to Pr [2, 19] and releases its repression of PIFs. *PIF4* expression is increased at hAT, and PIF4 activates the expression of temperature-responsive genes [20]. PIF4 is critical for temperature-induced morphological responses, including hypocotyl and petiole elongation and leaf hyponasty. These responses are absent in *pif4* mutant plants, except for early flowering [13, 21, 22]. Notably, *pif4* mutants fail to induce the expression of temperature-responsive genes, such as the auxin biosynthetic gene *YUCCA 8* (*YUC8*) and the brassinosteroid biosynthetic gene *DWARF 4* [9, 13, 20, 21]. It highlights the importance of PIF4 in regulating thermomorphogenesis.

Both hAT and heat shock affect different steps of plant reproduction, and consequently the production of viable seeds [23]. Heat shock impairs pollen viability and fertilization in pea [24], rice [25], and chickpea [26]. Heat shock reduces seed yield and quality in wheat [27], rice [28], and chickpea [29]. The long-term effects of hAT during reproduction have been studied in oilseed rape [30, 31] and a few hints of molecular pathways activated to cope with hAT have been listed [32, 33], including response to heat stress, ROS production, and photosynthesis.

We studied the effect of temperature below the stress threshold across all developmental stages and throughout the plant life cycle in *A. thaliana.* We focused on developmental processes that have not been thoroughly studied before, such as adult traits and reproduction organ formation. Through detailed phenotyping techniques of wild-type (Col-0) plants and phyB-PIF pathway mutant lines under normal (nAT) and high (hAT) ambient temperature conditions, we asked whether phyB and PIF4 are also part of the core mechanism that regulates temperature responses in these developmental processes, as they do in seedlings. This study uniquely combines multiple phenotyping approaches: seeds with Boxeed, ovules and embryos with microscopy, roots with Rhizotron, and plants with PlantScreen. We complemented this phenotyping by examining the transcriptomic response in pistils of wild-type, *phyB*, and *35 S::PIF4* plants grown in nAT and hAT to identify the potential regulatory pathways that might explain the reduced fertilization rate of the wild-type plants under hAT.

## Methods

### Plant materials and growing conditions

*Arabidopsis thaliana* seeds from Col-0 (own seed stock), homozygous mutant lines of *pif3-7* (*N66042*, obtained from NASC), *pif4-2* (*N66043*, *sail_1288_E07*, obtained from NASC), *pif7-1 (N68809*, obtained from NASC*)*,* pif7-2 (N71656*,* sail_622_G02*, obtained from NASC*)*,* pif3-3 pif7-1 (N68810*, obtained from NASC*)*,* pifq (N66049*, obtained from NASC; *pif1-1 (sail_256_G07) pif3-7 pif4-2 pif5-3 (N66044*,* salk_087012)*,* phyB-9 (N6217*, obtained from NASC), *35S::PIF4* (kindly provided by Zhi-Yong Wang, Carnegie Institution for Science, Stanford, USA), and *YUC4::3nGFP*, *YUC8::GUS-GFP*, and *TAA1::GFP-TAA1* reporter lines (own seed stock) were used for this study [34–39]. Seeds were sterilized with 20% bleah, washed twice in sterile distilled water, and stratified at 4 °C for 24 h. Plants were either germinated directly in soil (mixture of 2/3 peat moss Substrate 3 [Klasmann-Deilmann GmbH, Germany] and 1/3 vermiculite) or on plates containing MS medium. In plates, plants were grown for ten days at 21 °C with a 16-h light/8-h dark photoperiod and 150 µmol.m^− 2^.s^− 1^ LED illumination before transfer to soil. For all the measurements, plants were grown in a walk-in Fytoscope growth chamber (FS-WI, Photon Systems Instruments (PSI), Czech Republic) under growth conditions with a long-day regime (16 h light/8 h dark), LED illumination with an intensity of 150 µmol.m^− 2^.s^− 1^, and 35–45% humidity. For normal conditions (nAT), the temperature was set at 21 °C during the day and 18 °C at night. For high ambient temperature conditions (hAT), the temperature was set at 28 °C during the day and 24 °C at night.

### Root phenotyping

The rhizotrons (203 × 293 × 29.5 mm, height x width x depth) (PSI, Czech Republic) with a transparent glass plate and a light-protected black sheet cover were filled with cultivation substrate Klasmann TS-3 fine (Klasmann-Deilmann GmbH, Germany) and tilted at 45^o^ with the glass plate facing downwards. Seeds from wild-type Col-0, *pif4*,* phyB*, and *35S::PIF4* lines were sterilized, stratified, and randomly sown in the rhizotrons. Plants were grown in phytotron (PSI, Czech Republic) under long-day conditions with light intensity of 150 µmol.m^− 2^.s^− 1^ and 40–60% relative humidity. After ten days of plant cultivation in nAT, half of the rhizotrons continued in nAT, and the other half in hAT. The soil temperature was measured with a soil temperature sensor Pt1000 with datalogger Microlog T3 (Environmental Measuring Systems Ltd, Czech Republic). The soil temperature was about 1 °C less than the air temperature in all conditions. Regular root phenotyping was performed three times a week using the PlantScreen™ SC System (PSI, Czech Republic) [40] equipped with a bottom-side root imaging unit (GigE PSI BW − 12.36 megapixel camera with 1.1” CMOS sensor) with LED-based light source.

Experiments were conducted in triplicate, with the first replicate consisting of five biological replicates and the following two replicates consisting of eight biological replicates for each genotype/condition. Rhizotron weights were measured prior to watering, and an equal amount of water was added to each tray. Subsequent watering occurred after the system had lost the weight of the added water. Raw data were automatically stored and processed using the PlantScreen™ SC Root Tester software (PSI, Czech Republic). Parameters such as primary root length, lateral root density, and length of the four longest lateral roots were evaluated manually using ImageJ. The Relative Growth Rate (RGR) is calculated as follow: (lengthT2-lengthT1)/(T2-T1).

### Shoot phenotyping

For shoot phenotyping, we examined nine *A. thaliana* lines, including Col-0, *pif3*,* pif4*,* pif7-1*,* pif7-2*,* pif3 pif7*,* pifq*,* phyB*, and *35S::PIF4*, in two experimental conditions (nAT and hAT). Each experiment consisted of 18 replicates per line. After sterilization and stratification, seeds were directly sown in pots (70 mm × 70 mm × 65 mm, Poppelman TEKU, Germany) containing 65 g of freshly sieved soil (Substrate 2, Klasmann-Deilmann GmbH, Germany), watered with 10 ml of water per pot, and grown in nAT for 10 days. All plants were then transferred to a climate-controlled growth chamber (FS-WI, PSI, Czech Republic). Growth conditions for day/night temperature were set at 21/18 °C for nAT and 28/24 °C for hAT. At least 17 plants of each genotype were monitored daily for 50 days in nAT and 42 days in hAT. The phenotyping protocol included multiple analyses, including photosynthesis-related traits using kinetic chlorophyll fluorescence imaging, morphological traits using RGB imaging, and VNIR hyperspectral imaging for reflectance profiling (400–850 nm).

The PlantScreen™ Compact System [41] facilitated the daily transport of trays for phenotypic analyses on conveyor belts from the dark/light acclimation chamber to the light-isolated imaging cabinets and the weighing and watering station, where plants were automatically weighed and watered daily to maintain the soil at a relative water content of 44% feld capacity. Photosynthetic performance was assessed using a light curve protocol (as described in [41]), which quantified the rate of photosynthesis at four different photon irradiances with 60 s intervals of cool white actinic light at 140, 270, 410, and 540 µmol.m^− 2^.s^− 1^ corresponding to L1, L2, L3, and L4, respectively. Raw data were automatically processed using the PlantScreen™ Analyzer software (PSI, Czech Republic).

### Reproductive tissues and embryo phenotyping

Col-0, *pif4*,* pifq*, and *phyB* plants were analyzed to assess reproductive organs and seeds. After sterilization and stratification, all seeds were germinated and grown on plates for approximately two weeks. They were then transferred to soil and grown under nAT until the development of the first flowering bud. Half of the plants of each genotype were then grown in hAT until the end of their life cycle. Anthers were dissected from flower buds one day before anthesis, mounted in Alexander’s staining solution [42], incubated overnight at 50 °C, and observed under a Zeiss Axioscope light microscope. For the ovule analysis, flowers were emasculated one day before anthesis. Two days later, mature ovules (FG7 stage) were dissected from the pistil, stained with 10 µg/mL propidium iodide for five minutes on microscope slides, and observed under a Zeiss LSM 700 laser scanning confocal microscope. Seeds containing embryos of different developmental stages were isolated from the silique and cleared using the ClearSee method [43], stained with Renaissance SR2200, and observed with a Zeiss LSM 700 laser scanning confocal microscope.

### Confocal microscopy in ovules

For auxin biosynthetic gene expression pattern, flowers were emasculated one day before anthesis. Two days later, mature ovules (FG7 stage) were dissected from the pistil, stained with 10 µg/mL propidium iodide on microscope slides for five minutes. Fluorescent signals were observed using a ZEISS 700 microscope equipped with a 25x magnification objective. GFP imaging was performed using 488 nm laser lines.

### Dry seed phenotyping

Non-invasive seed phenotyping analysis was performed using the Boxeed robot (Labdeers, Czech Republic). The seed sorting mode was used to understand the distribution of individual phenotypic traits in the progeny of nAT and hAT grown plants. In this mode, 1,000 seeds were randomly analyzed in two biological replicates. The parameters of individual seeds were analyzed from two orientations with an angular position of the nozzle at 0° and 90°. Seed analysis was performed using the Boxeed software for various seed morphometric parameters, including the seed size (mm^2^), shape (ratio of seed length to area), length (mm), and width (mm). An average of two measurements for each seed was used to calculate seed characteristics.

### Data analysis

The results (multiple pairwise comparisons between different conditions and temperatures) were analyzed using Exact Fisher’s and two-way ANOVA followed by Tukey’s *post hoc* test using Python (Python Software Foundation, https://www.python.org/) in the Pycharm environment (https://www.jetbrains.com/pycharm/). The level of statistical significance was set at *p* ≤ 0.05 for all tests. Statistical analysis is described in the Additional File 2. Graphs for the representation of the phenotyping data were prepared using SuperPlotsOfData (https://huygens.science.uva.nl/SuperPlotsOfData/) [44].

The relative response to hAT for each tissue and parameter was evaluated as a percentage. The Pearson correlation method in Python was used to calculate the correlations between different parameters, resulting in a correlation matrix. To maintain the integrity of the analysis, a significance threshold of a *p*-value of 0.005 (0.5%) was set. This threshold ensured that only correlations with *p*-values below this level were considered significant and included in the graph. The resulting matrix provides valuable insight into the intricate relationships between different tissues and parameters in response to hAT.

### RNA extraction, library construction and RNAseq

Gynoecium samples from flowers at stages 11 and 12 (before anthesis) were collected from wild-type, *phyB* and *35S::PIF4* plants. Plants were cultivated as for the phenotyping of the reproductive organs: grown at nAT until the start of flowering, then kept at nAT or moved to hAT. Total RNA was extracted from 100 mg of pistils using the RNeasy Plant Mini Kit (Qiagen) following the manufacturer’s protocol. RNA isolates were treated with rDNAse (Macherey-Nagel) to remove traces of contaminant DNA and purified using a RNeasy MinElute Cleanup Kit (Qiagen). RNA quality was assessed using a NanoDrop2000 spectrophotometer and agarose gel electrophoresis. Samples, four biological replicates each, were sent to the Novogene Genomic Sequencing Labs (Cambridge Sequencing Center) for sequencing. All samples passed Novogene’s quality control threshold for library preparation and RNA-seq. mRNA-Seq libraries were constructed by Novogene, starting with 100 ng of high-quality RNA per sample. mRNA purification was performed using oligo(dT)-attached magnetic beads, followed by fragmentation and first-strand cDNA synthesis. Second-strand cDNA synthesis, end repair, adapter ligation, and size selection were performed. PCR enrichment yielded in the final cDNA library. Sequencing was conducted on the Illumina NovaSeq platform, generating 150-bp/150-bp paired-end reads. The sequence data have been deposited in the Genbank database under the BioProject PRJNA1091589 (https://www.ncbi.nlm.nih.gov/bioproject/PRJNA1091589). Clean reads were generated by removing adaptor sequences and low-quality reads using fqtools. The reads were mapped to the *Arabidopsis* genome using Araport11 (TAIR10, http://www.arabidopsis.org/). FeatureCounts was used to determine read count for each gene in each sample. The FPKM values were calculated to provide a measure of gene expression levels in each sample.

### Differential gene expression (DE) analysis

Differential gene expression analysis was analyzed by Bioconductor package DESeq2 v1.34.0 [45]. Data generated by DESeq2 with independent filtering were selected for the differential gene expression analysis due to its conservative features and to avoid potential false positives. Genes were considered to be differentially expressed based on a cut-off of adjusted *p*-value < 0.05 and log2(fold-change) ≥ 1 or ≤-1 and a false discovery rate (FDR) < 0.05.

### Gene Ontology and hierarchical clustering

Gene ontology annotation was retrieved from EnsemblPlants, Ensembl BioMarts [46]. Gene enrichment was performed using the R package clusterProfiler [47] on the differentially expressed genes (genes with adjusted *p*-value < 0.05) and separated in up- and down-regulated set. Visualizations were made using ggplot2 [48]. Hierarchical clusters were generated from selected top differentially regulated genes using R package pheatmap v1.0.12 1, volcano plots were produced using ggplot2 v3.3.5 package [48] and MA plots were generated using ggpubr v0.4.0 package 2.

## Results

### High ambient temperature alters the root system architecture with a reduction in the number of emerged lateral roots compensated by their increased elongation

**Fig. 1:**
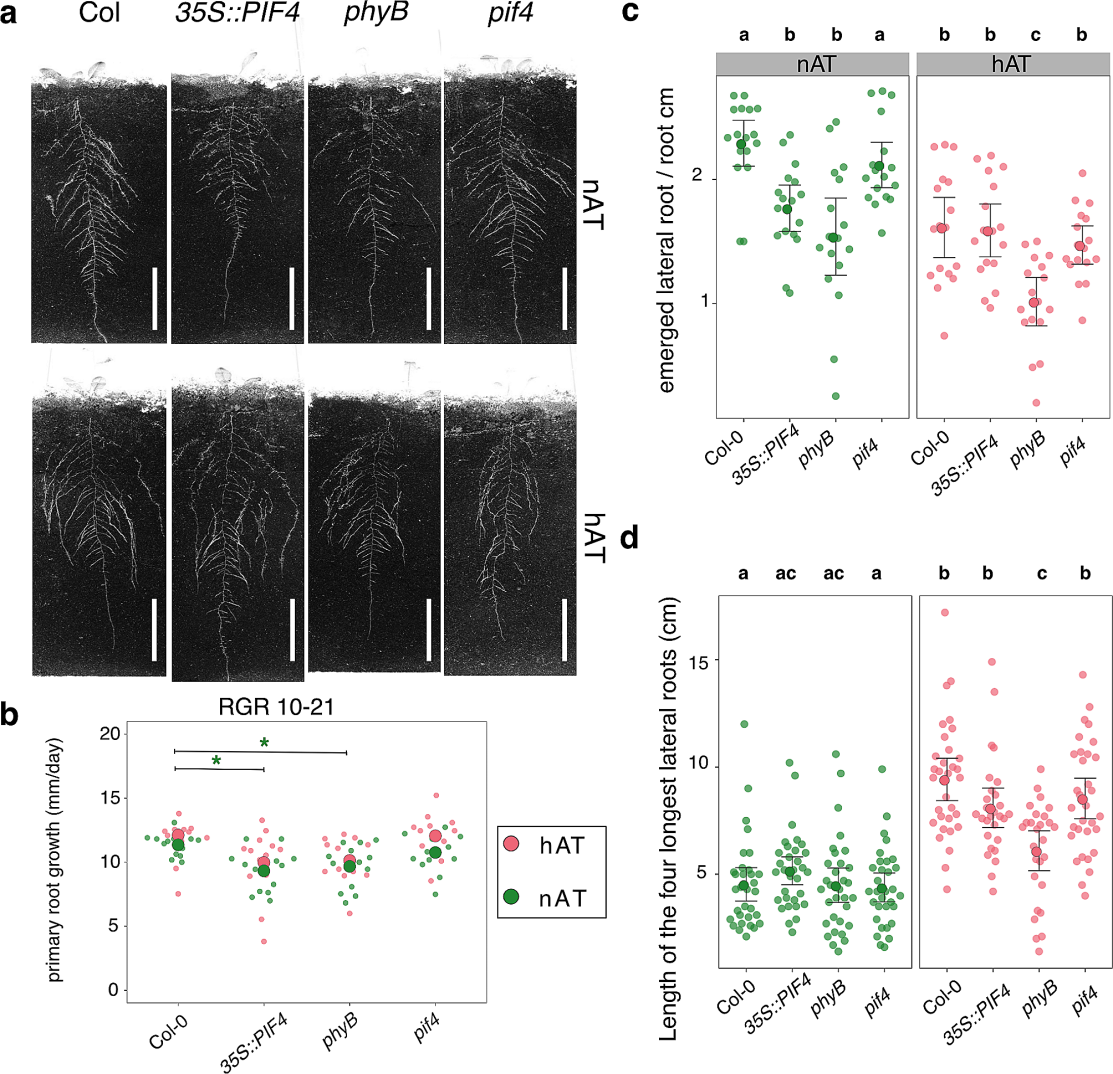
Temperature suppresses lateral root formation and promotes lateral root elongation. (**a**) Root morphological phenotype of 21-day-old plants of Col-0, *35S::PIF4*, *phyB*, and *pif4* at nAT (top row) and hAT (bottom row). Scale bars represent 5 cm. (**b**) Relative growth rate (RGR) of the primary root (in mm per day) between 10 and 21 days after. Data for the primary root length over time in nAT and hAT are shown in Additional File 1 – Fig. S1. (**c**) Quantification of lateral root density, expressed as the number of lateral roots per centimeter of the primary root for each genotype. *n* = 16 plants per genotype per condition in triplicate. (**d**) Length of the four longest lateral roots (in cm) at maturity. *n* = 16 plants per genotype. Data for nAT in green and hAT in red. Statistical analysis and data source are provided in Additional File 3 – Tables S1 and S2. (**b**) The significant effects of the temperature are depicted as *. The *p*-value ranges are specified as * for *p*-values between 0.05 and 0.01, ** for *p*-values between < 0.01 and 0.0001, *** for *p*-values lower than 0.0001. The color of the * matches the color of the temperature (green for nAT and red for hAT). (**c**, **d**) Genotypes that share the same letter are not statistically significantly different

Roots developing in darkness are different from roots growing in light, and this also affects how they respond to temperature [49, 50]. Therefore, we used a light-isolated rhizotron system that allows plants to grow in natural conditions for non-invasive, image-based root phenotyping. To investigate the effects of temperature on root morphology and the possible involvement of the phyB-PIF4 pathway, we phenotyped Col-0, *phyB*, *35S::PIF4*, and *pif4* plants. Lateral and adventitious roots, total root length, primary root length, maximum length of the four longest lateral roots, and root area were measured throughout growth. Root growth patterns responded differently to warm temperatures among genotypes (Fig. 1a). The relative growth rate and length of the primary root were not affected at hAT compared to nAT (Fig. 1b; Additional File 1 – Fig. S1; Additional File 3 – Tables S1, S2), consistent with previous work using a TGRooZ device that mimics natural conditions [50]. However, both *phyB* and *35S::PIF4* displayed a shorter final primary root length under both conditions, significantly shorter only at nAT (Fig. 1b; Additional File 3 – Table S2). Lateral root formation was inhibited at hAT. Wild-type, *phyB*, and *pif4* plants showed reduced lateral root density (number of emerged lateral roots per cm of primary root) at hAT. However, lateral root density was not affected in *35S::PIF4* plants at hAT compared to nAT (Fig. 1c; Additional File 2 – Table S1; Additional File 3 – Table S2). At hAT, the number of lateral roots in wild-type plants was similar to that of *phyB* and *35S::PIF4* at nAT. Although the plants had fewer emerged lateral roots, hAT promoted their elongation in all the genotypes; expect for *phyB* (Fig. 1d). There may be a trade-off between the number of lateral roots and their length. All the genotypes increased the average length of the four longest lateral roots with Col-0 and *pif4* seedlings being the most affected and *35S::PIF4* and *phyB* being the least sensitive to hAT (Fig. 1a and d; Additional File 2 – Table S2; Additional File 3 – Table S2). The opposite effects of hAT on the number of lateral roots and their length did not significantly affect the total root length and root area between nAT and hAT (Additional File 1 – Figs. S2 and S3). However, these values were significantly lower for *phyB* and *35S::PIF4* genotypes under both conditions, resulting in a reduced root system compared to wild-type plants. Furthermore, the temperature increase promoted the induction of adventitious roots in all the genotypes studied. 17.64% of the wild-type and *phyB* plants produced adventitious roots at hAT, while this value decreased to 5.88% for the *PIF4*-modified genotypes (Additional File 3 – Table S2). These changes in (lateral) root length and number alter the root system architecture of plants grown at hAT. We observed that *phyB* and *35S::PIF4* plants under both conditions have a reduced root system compared to wild-type plants (Additional File 1 – Figs. S2 and S3), and that the *phyB* mutants at nAT and wild-type plants under hAT have a similar lateral root density (Fig. 1d). These results suggest that hAT causes alterations in root architecture by decreasing phyB activity, as proposed for hypocotyl growth.

### Repression of *phyB* mimics the effects of high ambient temperatures on *Arabidopsis* shoot architecture

**Fig. 2:**
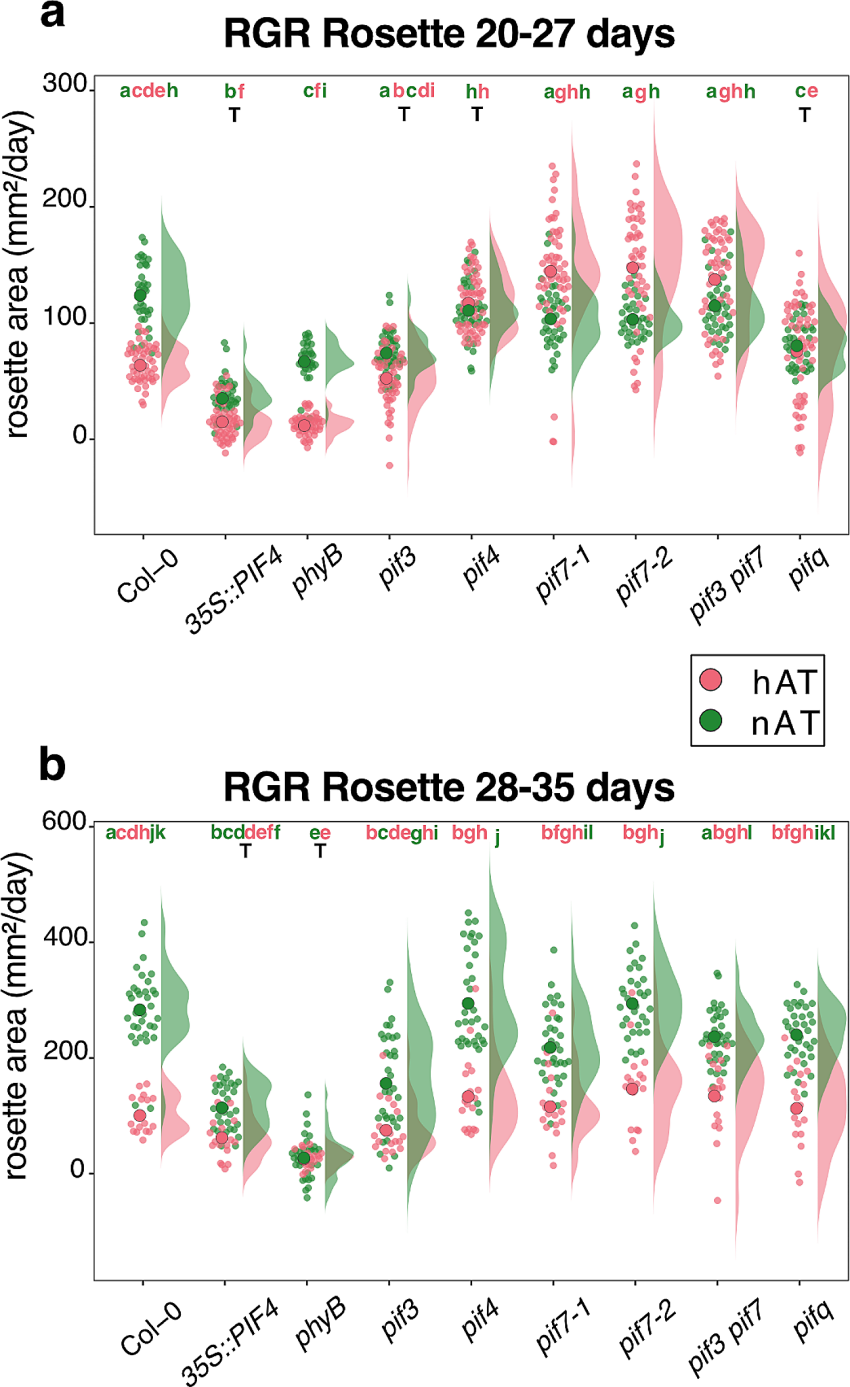
Temperature-induced reduction in rosette area. (**a**,** b**) Relative growth rate of the rosette area from 20 to 27 days after sowing (**a**) and from 28 to 35 days after sowing (**b**). Green represents data for nAT and red for hAT. Data source and statistical analysis are provided in Additional File 3 – Tables S3 and S4. Plants are presented in Additional File 1 – Fig. S4. The time series for individual genotypes is presented in Additional File 1 – Fig. S5. Genotypes that share the same letter are not statistically significantly different. The color of the letter matches the color of the temperature (green for nAT and red for hAT). The letter T indicates that there is no statistical effect of the temperature

To investigate how hAT affects shoot development and explore the possible participation of the phyB-PIF4 pathway, we studied eight lines: *35S::PIF4*,* phyB*,* pif3*,* pif4*,* pif7-1*,* pif7-2*,* pif3 pif7*, and *pifq (pif1 pif3 pif4 pif5*). We quantified the effects of hAT on plant growth by measuring the rosette area from 9 to 39 days after sowing, when the plants reached their final rosette size (Fig. 2, Additional File 1 – Figs. S4, S5). The *phyB* and *35S::PIF4* plants exhibited delayed rosette expansion, starting at 26 days after sowing, while the other genotypes expanded from 22 days after sowing (Fig. 2a, Additional File 1 – Fig. S5). At nAT, wild-type plants had the largest area (40 cm²), whereas *35 S::PIF4* and *phyB* plants were smaller (20 cm² and 10 cm², respectively) (Additional File 1 – Figs. S4, S5). Other genotypes (*pif3*,* pif7*, and *pifq*) produced plants with intermediate rosette areas. This is a consequence of a significant reduced growth rate between 20 and 27, and between 28 and 33, days after sowing in *35S::PIF4* and *phyB* plants. It is noteworthy that the *phyB* plants stopped expanding after 27 days (Fig. 2b; Additional File 1 – Fig. S5). Wild-type plants were significantly sensitive to hAT, with a reduced growth rate and a final area of 15 cm² (Additional File 1 – Fig. S5). In contrast, *35S::PIF4*, *pif3*, *pif4*, and *pifq* maintained a stable growth rate between 20 and 27 days (Fig. 2a) but *pif3*, *pif4*, and *pifq* slowed down their growth rate after 28 days (Fig. 2b). The final rosette area at hAT was about 20 cm² for *pif4*,* pif7-1*,* pif7-2*, and *pif3 pif7* plants. The *phyB* and *35S::PIF4* plants showed the smallest area with only 5 cm². The wild type, *pif3*, and *pifq* showed an intermediate size of 15 cm² (Additional File 1 – Fig. S5).

To analyze the effects of hAT on inflorescence architecture, we counted the number of inflorescence stems (primary and lateral) emerging from rosettes in all genotypes under both growth conditions. While most genotypes produced an average of five inflorescence stems under normal conditions, *phyB* and *35S::PIF4* plants produced an average of three inflorescence stems. When exposed to hAT, stem production decreased in almost all genotypes, two stems for *phyB* and *35S::PIF4* plants, and three stems for the other genotypes Wild-type plants under hAT mirrored the performance of *phyB* and *35S: PIF4* under nAT. Notably, the *pif3 pif7* plants appeared to be resilient to the hAT, producing an average of 4 stems (Table 1).

**Table 1:** Number of primary inflorescence stems for different genotypes at nAT and hAT

Genotype	Condition	Average number of stems	SE	Statistical groups
Col_0	nAT	4.7	0.152	a
*phyB*	nAT	3	0.100	b, c
*35S::PIF4*	nAT	3.5	0.166	b, d
*pif3*	nAT	4.4	0.276	a
*pif4*	nAT	5.3	0.276	a
*pif7-1*	nAT	5.3	0.221	a
*pif7-2*	nAT	4.9	0.163	a
*pif3 pif7*	nAT	4.8	0.266	a, e
*pifq*	nAT	4.7	0.314	a
Col_0	hAT	3.1	0.100	c
*phyB*	hAT	2.1	0.298	d
*35S::PIF4*	hAT	2.1	0.133	d
*pif3*	hAT	3.7	0.200	c
*pif4*	hAT	3.7	0.221	c
*pif7-1*	hAT	4.5	0.339	e
*pif7-2*	hAT	4	0.314	c
*pif3 pif7*	hAT	4.3	0.213	e
*pifq*	hAT	3.6	0.163	c

*n* = 10 plants per genotype were analyzed to assess differences among genotypes and between nAT and hAT using a two-way ANOVA. Post-hoc Tukey’s test identified non-significant differences between genotypes with the same letter

**Fig. 3:**
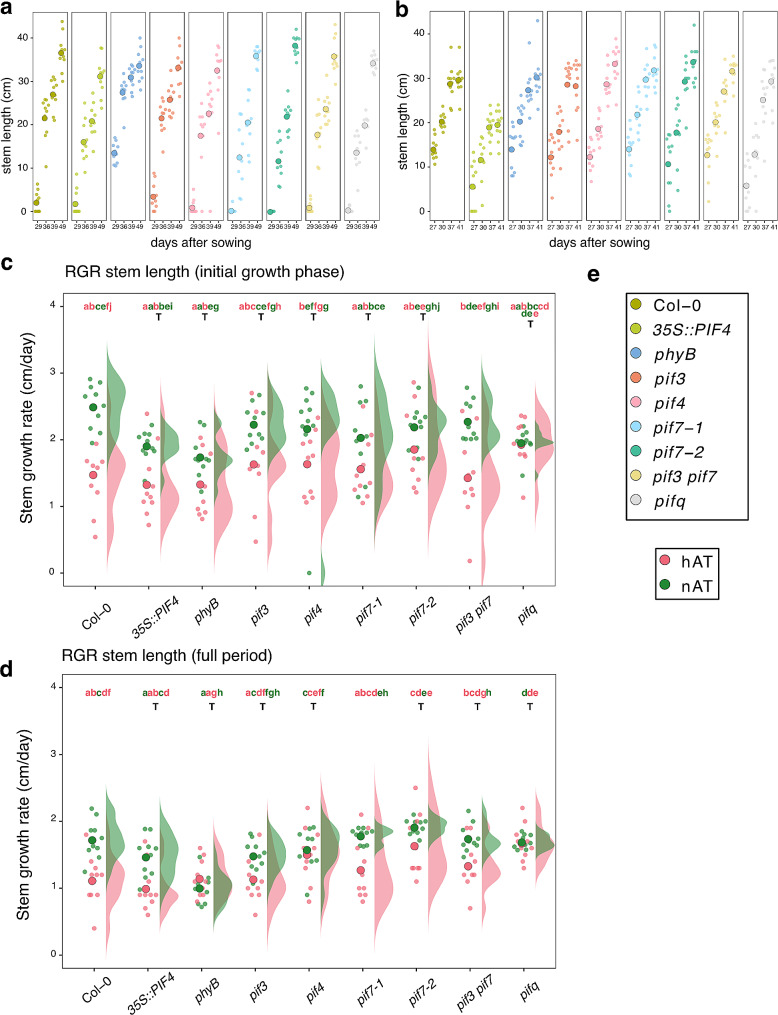
Temperature-induced early flowering and decreased inflorescence stem length. (**a**,** b**) Time series of the progression of primary inflorescence stem elongation (in cm) from 20 to 49 days after sowing for nAT (**a**) and up to 41 days after sowing for nAT (**b**). (**a**, **b**) *n* = 10 plants per genotype per condition. (**c**,** d**) Relative growth rate of the stem (in cm/day) during the full measurement period (29 days to 49 days after sowing at nAT, and 27 days to 41 days after sowing at hAT) (**c**) and from 29 to 39 days after sowing for nAT or from 27 and 27 days for hAT (**d**). (**e**) Color legend for a-d is provided. Data source is provided in Additional File 3 – Tables S3 and S5. Plants are presented in Additional File 1 – Fig. S4. The color of the letter matches the color of the temperature (green for nAT and red for hAT). The letter T indicates that there is no statistical effect of the temperature

The inflorescence growth pattern was affected in hAT (Fig. 3; Additional File 1 – Fig. S4). In nAT, the first flowers of the primary inflorescence stem opened between 25 and 29 days in *phyB* and between 29 and 36 days in the other genotypes. Consequently, *phyB* primary inflorescence stems were longer than Col-0 stems during their growth period, e.g., until 36 days, when both genotypes reached a comparable height (averaging 35 cm by the last observation point at 49 days for all genotypes (Fig. 3a)). However, the growth rate of the *phyB* primary inflorescence stem was significantly lower than that of the wild-type stem (Fig. 3c and d; Additional File 3 – Table S3). The *phyB* mutant also stopped flowering earlier, at 44 days, while the other genotypes continued flowering until 49 days (Fig. 3a). hAT stimulated early initiation of inflorescence stem elongation in all genotypes at 23 days, similar to that observed in *phyB* plants grown under nAT (Fig. 3a and b). In the primary inflorescence stem, flowers opened around 27–30 days in hAT. Plants reached their maximum growth earlier, at 41 days, supported by the significantly reduced growth rate in hAT in wild-type, *pif7-1*, and *pif3 pif7* plants (Fig. 3c and d; Additional File 3 – Table S3). This resulted in a shorter final height ranging from 13 to 38.9 cm (*35S::PIF4* stems being the shortest) at hAT, while this value corresponds to 18–43.8 cm at nAT (Fig. 3a and b). The main inflorescence stem growth rate was insensitive to temperature changes throughout the entire flowering period in *phyB*, *35S::PIF4*, *pif3*, *pif4*, *pif7-2*, *pif3 pif7*, and *pifq* plants (Fig. 3d), and only at the start of the flowering period in *pif7-1* plants (Fig. 3c). These results indicate that hAT influences above-ground vegetative growth by reducing shoot expansion and branching. hAT prioritizes flowering over vegetative growth. At nAT, the *phyB* mutant mimics the patterns observed in wild type under hAT, suggesting that phyB may participate in these thermomorphogenic processes.

### Ambient temperature has a moderate impact on plant fitness but modulates photosynthetic parameters

To know if the developmental alterations caused by hAT are linked to changes in the energy status of the plants, we examined several parameters commonly associated with photosynthetic performance: reflectance profile and pigment content. Hyperspectral imaging in the visible and near infrared (350–900 nm wavelength, VNIR) measures the light reflectance of plant leaves. It is an important indicator of plant fitness status [51, 52]. In our study, we measured VNIR parameters, including the Normalized Difference Vegetation Index (NDVI), Optimized Soil-Adjusted Vegetation Index (OSAVI), Photochemical Reflectance Index (PRI), Modified Chlorophyll Absorption Ratio Index 1 (MCARI1), Structure Insensitive Pigment Index (SIPI), and Plant Senescence Reflectance Index (PSRI).

In nAT, NDVI increased with age until 29 days after sowing for all genotypes and remained stable until the end of the measurements at 34 days (Additional File 1 – Fig. S6a; Additional File 3 – Table S6). hAT reduced the NDVI in all genotypes ranging from 0.68 to 0.78, especially at later growth stages (22–28 days after sowing) (Additional File 1 – Fig. S6a). In both nAT and hAT, NDVI had lower values for *phyB* and *35S::PIF4* with values in nAT (an average of 0.74) being comparable to NDVI values (an average of 0.82) of the other genotypes in hAT. OSAVI, which is designed to mitigate the effects of soil on NDVI, mirrored the trends observed in NDVI (Additional File 1 - Fig. S6b; Additional File 3 – Table S6). These two parameters are indicators of plant vegetative fitness [53, 54]. Therefore, it can be concluded that both the repression of phyB activity and hAT affect the vegetative vitality of the plant.

PRI and PSRI parameters were mostly not significantly affected by the different ambient temperatures for all genotypes (Additional File 1 – Figs. S6c, S6d; Additional File 3 – Table S6). PRI values decreased with the plant age, whereas the opposite was observed for PSRI, which measures plant senescence based on the ratio of carotenoids to chlorophyll. Again, *phyB* and *35S::PIF4* plants had lower PSRI values than wild type at nAT and hAT. The SIPI parameter is sensitive to chlorophyll and carotenoid content [55] and MCARI1 parameter is associated with the chlorophyll content in plant leaves [56]. Both values increased as the plants aged at nAT and hAT (Additional File 1 – Figs. S6e, S6f; Additional File 3 – Table S6). All other genotypes, except *35S::PIF4* and *phyB*, had reduced SIPI values at hAT. The *35S::PIF4* and *phyB* plants had lower SIPI values at nAT and did not respond to hAT. A similar trend was observed for the MCARI1 parameter.

We applied chlorophyll fluorescence imaging to assess the efficiency of the plants to use the light energy for photosynthesis in the studied genotypes at nAT and hAT. The parameter QY-max (F_V_/F_M_) indicates the maximum quantum efficiency of the photosystem II (PSII) photochemistry. QY-max of wild-type plants increased steadily with age, with values ranging from 0.79 to 0.84 for nAT and 0.79–0.82 for hAT (significant difference only between 14 and 32 days after sowing). In nAT, the QY-max values for *phyB* and *35S::PIF4* plants were lower than in the wild type. Interestingly, *phyB* recovered to wild-type QY-max values after two weeks of cultivation at nAT (Additional File 1 – Fig.S7a). At hAT, QY-max values increased with age for all genotypes, except *35S::PIF4* and *pif3* (Additional File 1 – Fig. S7a). Photosynthetic efficiency was also measured in light-adapted plants. In particular, the parameters QY-Lss (PSII operating efficiency), and qP (photochemical quenching coefficient) [57] displayed significantly higher values at hAT for all the genotypes, corresponding to those of 39-day-old plants grown at nAT for both low and high light saturation point (Lss1 and Lss4) (Additional File 1 – Fig. S7c–f). For the two light intensities at hAT, the age of the plants did not impact the values of the two parameters. Non-photochemical quenching (NPQ) assesses the damage to photosystems caused by various environmental stressors [58]. All the genotypes exhibited lower NPQ values at hAT, indicating the negative impact of the high ambient temperature on the photosystem activity (Additional File 1 – Figs. S7g, S7h). Compared to the wild type, the *phyB* and *35S::PIF4* plants showed elevated NPQ values at nAT and hAT. Overall, those parameters indicate that hAT and the repression of phyB reduces plant fitness and photosynthesis efficiency.

**Fig. 4:**
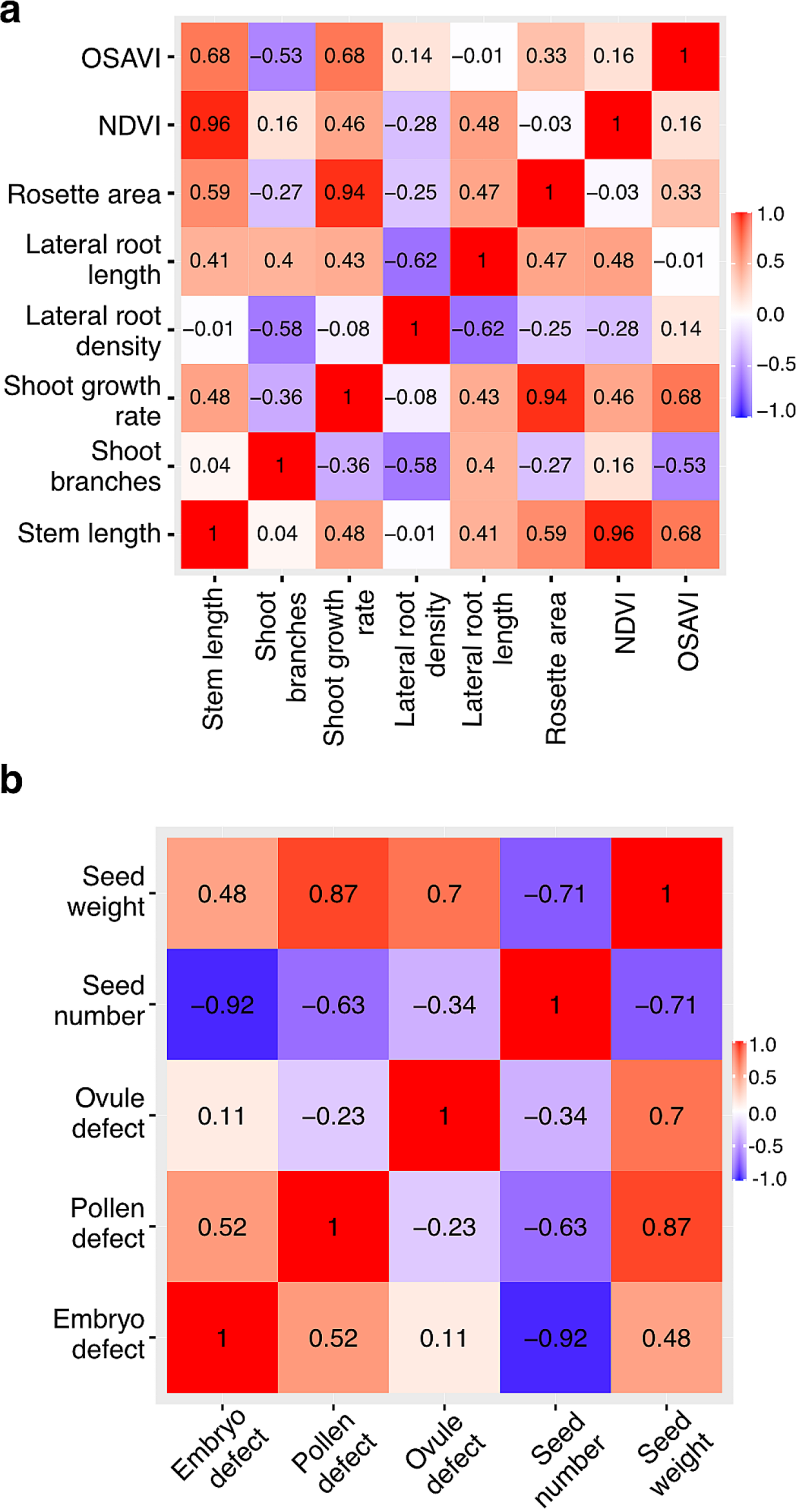
Correlation of the hAT response in vegetative and reproductive tissues. Correlative analysis of the response to hAT in vegetative (**a**) and reproductive (**b**) tissues for the different measured parameters

Given that hAT affects multiple processes through phyB-PIF in shoot and root, we questioned whether these processes are independent, co-regulated or indirect consequences of other primary effects. To investigate these possibilities, we performed correlation analysis between all traits observed in Col-0 (Fig. 4). These matrices display correlations with *p*-values below the significance threshold of 0.05, indicating statistically significant relationships between the relative responses to hAT in the different organs. During vegetative growth (Fig. 4a), a positive correlation (0.96) was observed between the NDVI parameter and the length of the inflorescence stem, highlighting the effectiveness of the NDVI parameter in indicating growth dynamics. A robust positive correlation (0.94) was also noted between inflorescence stem growth rate and rosette area for their response to hAT. Notably, a negative correlation (-0.62) was observed between lateral root density and lateral root length, hinting at a potential trade-off mechanism governing root development.

### *phyB* influences the response of reproductive tissues to hAT

We have used Col-0, *phyB*, *pif4*, *pifq*, and *35S::PIF4* plants to investigate whether the phyB-PIF4 pathway regulates thermomorphogenesis during reproductive development, focusing in anthers and mature ovules. To ensure a similar fitness of the plants at the reproductive stage, plants were exposed to hAT after the first flower bud appearance and maintained at hAT until the end of their growth.

Anthers were collected at 7 and 9 days after the development of the first flower (DAFD) on the primary inflorescence stem. In nAT, we did not observe any abnormality in the different lines. In hAT, the wild type, *phyB*, and *35S::PIF4* lines were affected to different degrees. At 7 DAFD, 4.65% of the wild-type anthers were aborted, while this percentage reached 23.40% and 11.43% for the *35S::PIF4* and *phyB* lines, respectively. Interestingly, these percentages increased to 7.81%, 29.27%, and 34.82%, respectively, at 9 DAFD when plants were subjected to prolonged hAT. Notably, only the *phyB* mutant showed a highly significant increase in this trend (Table 2; Additional File 2 – Table S3). This observation suggests that the *phyB* plants may become increasingly sensitive to hAT as they progress through later developmental stages. Additionally, we observed that *pif4* and *pifq* anthers were more resistant to hAT than wild type, with abortion rates of only 1.11% and 1.44%, respectively, at 9 DAFD. Our results suggest that repression of phyB, resulting in PIF4 activation, worsens the negative effect of hAT on anther development.

**Table 2:** Anther abortion rate for different genotypes in nAT and hAT

			7 DAFD				9 DAFD			
		**Defective**		**Normal**	**n**	**% Defects**	**Defective**	**Normal**	**n**	**% Defects**
Col-0 nAT		0		57	57	0	0	68	68	0
Col-0 hAT		2		41	43	4.65	5	59	64	7.81 *
*phyB* nAT		0		93	93	0	0	87	0	0
*phyB* hAT		4		31	35	11.43 *	39	73	112	34.82 *** ### ^^^
*35S::PIF4* nAT		0		87	87	0	0	92	0	0
*35S::PIF4* hAT		11		36	47	23.40 *** #	12	29	41	29.27 *** ##
*pif4* nAT		0		89	89	0	0	106	0	0
*pif4* hAT		0		90	90	0	1	89	90	1.11
*pifq* nAT		0		103	103	0	0	91	91	0
*pifq* hAT		0		81	81	0	2	137	139	1.44 #

The anthers were assessed at 7 and 9 days after flowering development (DAFD). Fisher’s Exact Test analyzed comparisons, with anthers from each genotype and condition examined across three replicates for result reliability. Significance indicators are: * (temperature), # (genotype), and ^ (time). P-values are represented as: * # (0.05 − 0.01), ## (< 0.01-0.0001), and *** ### ^^^ (< 0.0001). Details are provided in Additional File 2 – Table S3

**Fig. 5:**
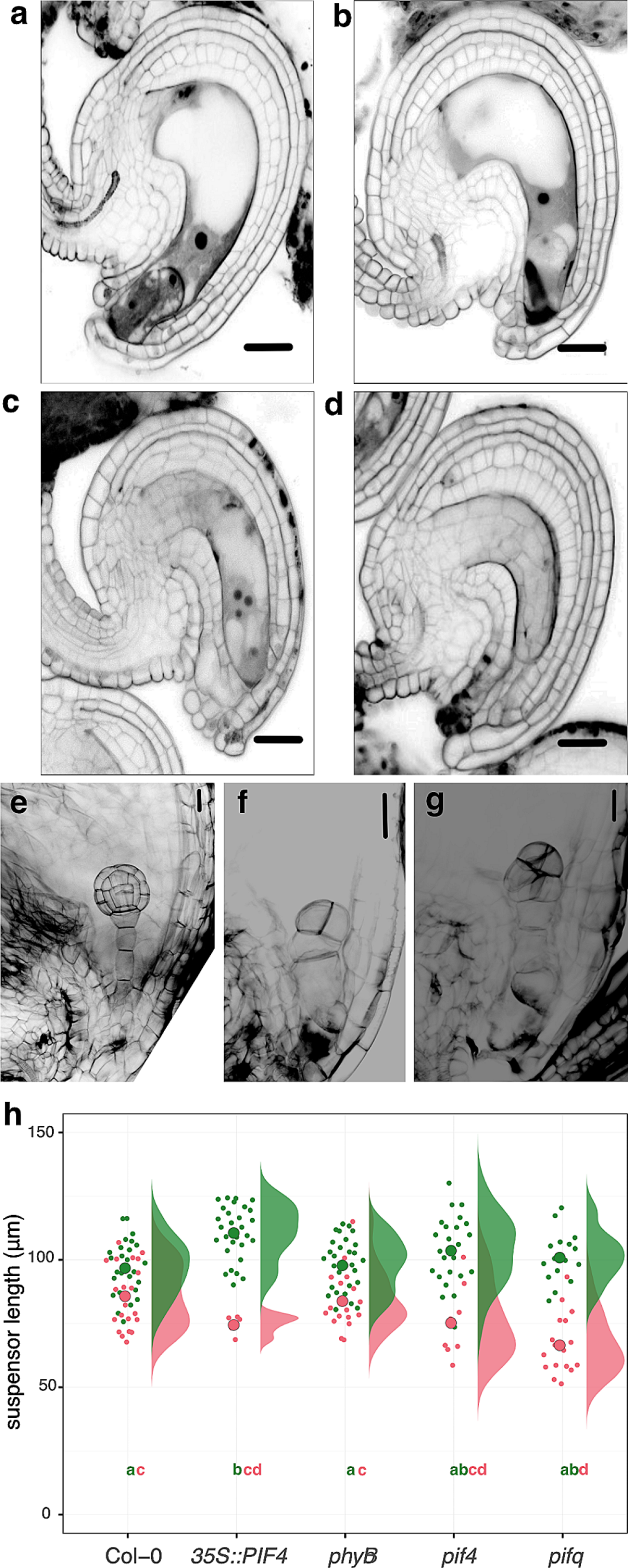
Effects of hAT on ovules and embryo patterning. (**a**-**d**) Representative pictures of the ovule phenotypes observed at the FG7 developmental stage observed at nAT and hAT in all genotypes: (**a**) normal ovule observed at nAT with the egg cell, one visible synergid cell and the fused nuclei in the central cell, (**b**) ovule with a collapsed synergid (black mass), (**c**) ovules with unfused central cell nuclei, and (**d**) ovule with a collapsed embryo sac. Scale bars represent 20 μm. The quantification of the phenotypes is provided as Table 3 and Additional File 2 – Table S4. *n* > 50 ovules per genotype per each condition, in triplicate. (**e**-**g**) Representative pictures of the embryo phenotypes observed at nAT and hAT in the seeds of the different genotypes: normal embryo (**e**), embryo with a dwarf suspensor (**f**), embryo exhibiting excessive cell divisions within the proper embryo region (**g**). Scale bars represent 20 μm. (**h**) Quantification of the suspensor length at nAT and hAT. n = appr. 20 suspensors per genotype per condition in triplicates. The quantification of the phenotypes is presented in Additional File 2 – Table S6. Genotypes that share the same letter are not statistically significantly different. The color of the letter matches the color of the temperature (green for nAT and red for hAT)

The same plants were analyzed to determine the effect of hAT on ovules. In nAT, 17.9% and 16.1% of *phyB* and *35S::PIF4* ovules, respectively, were defective, whereas the other lines had between 5.4% and 9.4% defective ovules (Fig. 5; Table 3; Additional File 2 – Table S4). Notably, only *phyB* and *35S::PIF4* lines were defective in the fusion of the central cell nuclei in nAT (Fig. 5c; Additional File 2 – Table S4). At hAT, all genotypes exhibited the same types of defects, predominantly a collapsed embryo sac (lacking synergid, egg cell, and central cell structures), collapsed synergids, and unfused central cell nuclei (Fig. 5a-d). Although the types of ovule defects were consistent across genotypes, the percentage of these defects varied (Additional File 2 – Table S4). *35S::PIF4* and *phyB* ovules were hypersensitive to hAT, producing 84.3% and 62.6% defective ovules, respectively (Table 3). In contrast, these percentages were only 30.6% and 27.6% in the wild-type and *pif4* lines, respectively. Interestingly, more ovules (45.9%) were defective in *pifq* than in *pif4* (27.6%), suggesting that other PIFs (such as PIF3, PIF5, or PIF7) may play a synergistic role in this response in ovules. We observed that repressing *PHYB* expression mimics the temperature effects observed in the wild type during ovule development, leading to the hypothesis that hAT alters ovule development by decreasing phyB activity.

**Table 3:** **Ovule defective phenotypes for the different genotypes at nAT and hAT**

Genotype	Growth conditions	Normal	Defective	*n*	% of defects	*p*-values
Col-0	nAT	122	7	129	5.4	
Col-0	hAT	102	45	147	30.6	***
*phyB*	nAT	78	17	95	17.9	#
*phyB*	hAT	31	52	83	62.6	*** ###
*35S::PIF4*	nAT	115	22	137	16.1	##
*35S::PIF4*	hAT	16	86	102	84.3	*** ###
*pif4*	nAT	90	8	98	8.2	
*pif4*	hAT	55	21	76	27.6	**
*pifq*	nAT	77	8	85	9.4	
*pifq*	hAT	37	31	68	45.6	*** #

The data per phenotype categories are detailed in Additional File 2 – Table S4. The statistical analysis of the data utilized Fisher’s Exact Test (* compared hAT vs. nAT and # compared the mutant vs. wild type). To ensure the reliability of our results, ovules from each genotype and condition were examined across three replicates. The significance levels in the results are denoted as follows: * significant temperature effect. # significant genotype effect. The *p*-value ranges are specified as # for *p*-values between 0.05 and 0.01, ** ## for *p*-values between < 0.01 to 0.0001, and *** ### for *p*-values lower than 0.0001

### An overlapping transcriptional response is observed between hAT wild-type pistils and nAT-grown *phyB* and *35S::PIF4* pistils

To better understand what would be the molecular mechanism behind the physiological response of *Arabidopsis* ovules to hAT, we performed a transcriptomic analysis of the gynoecium from 7 DAFD flowers at stage 11–12 (pre-anthesis, ovules at FG7) of Col-0, *phyB* and *35S::PIF4* plants. Plants were grown under nAT until the start of flowering (as described for the phenotyping of reproductive structures), and either kept at nAT or transferred to hAT after the start of flowering. More than 40 million reads were obtained from each sample (Additional File 2 – Table S5), with an average of 45% GC content. RNA-seq data received a high quality score by the Phred of 98 for Q20 and 94 for Q30 in average.

In comparison with nAT, the wild-type pistils under hAT had 8,485 differentially expressed genes (DEGs) (5,032 up-regulated and 3,453 down-regulated). While *phyB* and *35S::PIF4* pistils at hAT exhibited lower numbers of DEGs compared to nAT: 1,862 and 2,612 genes, respectively, with 1,037 and 2,062 genes up-regulated, and 825 and 550 genes down-regulated, respectively (Additional File 1 – Fig. S8a; Additional File 4 – Table S1).

The phenotyping analysis of the ovules indicated that the *phyB* and *35S::PIF4* plants at nAT behaved as Col-0 at hAT. However, a Principal Component (PC) analysis of all RNAseq pistil samples (Additional File 1 – Fig.S9) indicated that while all samples from nAT grouped together, the wild-type pistils under hAT isolated from all other samples (PC2, 53.15%) and the pistils from the *phyB* and *35S::PIF4* plants at hAT (PC1 17.39%), suggesting that wild-type and mutant pistils have a unique transcriptional behavior in response to hAT. Therefore, we compared the DEG patterns of the wild-type pistils in response to hAT (hAT vs. nAT) with those of *phyB* and *35S::PIF4* pistils at nAT (mutant nAT vs. wild type nAT). The number of up- and downregulated DEGs in these comparisons was very similar (Additional File 1 – Fig. S8b; Additional File 4 – Table S1). Venn diagrams analyze the overlap of the up and down DEGs in the same comparisons. In comparison with nAT, 10% (542 genes) of the upregulated genes from wild-type pistils under hAT were also upregulated in *35S::PIF4* and *phyB* pistils at hAT, whereas only 3% (121 genes) of the downregulated genes from wild-type pistils at hAT were also downregulated at hAT in the two mutants (Additional File 1 – Figs. S8c, S8d; Additional File 4 – Table S1). The majority of the upregulated DEGs (61.3%) in wild-type pistils at hAT vs. nAT were also found to be upregulated in *35S::PIF4* pistils at nAT vs. wild-type pistils at nAT (Additional File 1 – Fig. S8e; Additional File 4 – Table S1). In addition, almost half of the genes downregulated in the wild-type pistils at hAT vs. nAT (46%) were downregulated genes in *phyB* and *35S::PIF4* pistils at nAT (compared to wild type at nAT) (Additional File 1 – Fig. S8f; Additional File 4 – Table S1). Wild-type *Arabidopsis* pistils (and ovules) developed at hAT showed pronounced transcriptional changes with a substantial overlapping regulation with *phyB* and *35S::PIF4* pistils developed at nAT. This suggests that the *Arabidopsis* response to hAT during pistil development may involve signaling pathways dependent on the phyB and PIF regulators.

### Gene ontology analysis identified biological processes affected by hAT and phyB-PIF4 signalling in pistils

**Fig. 6:**
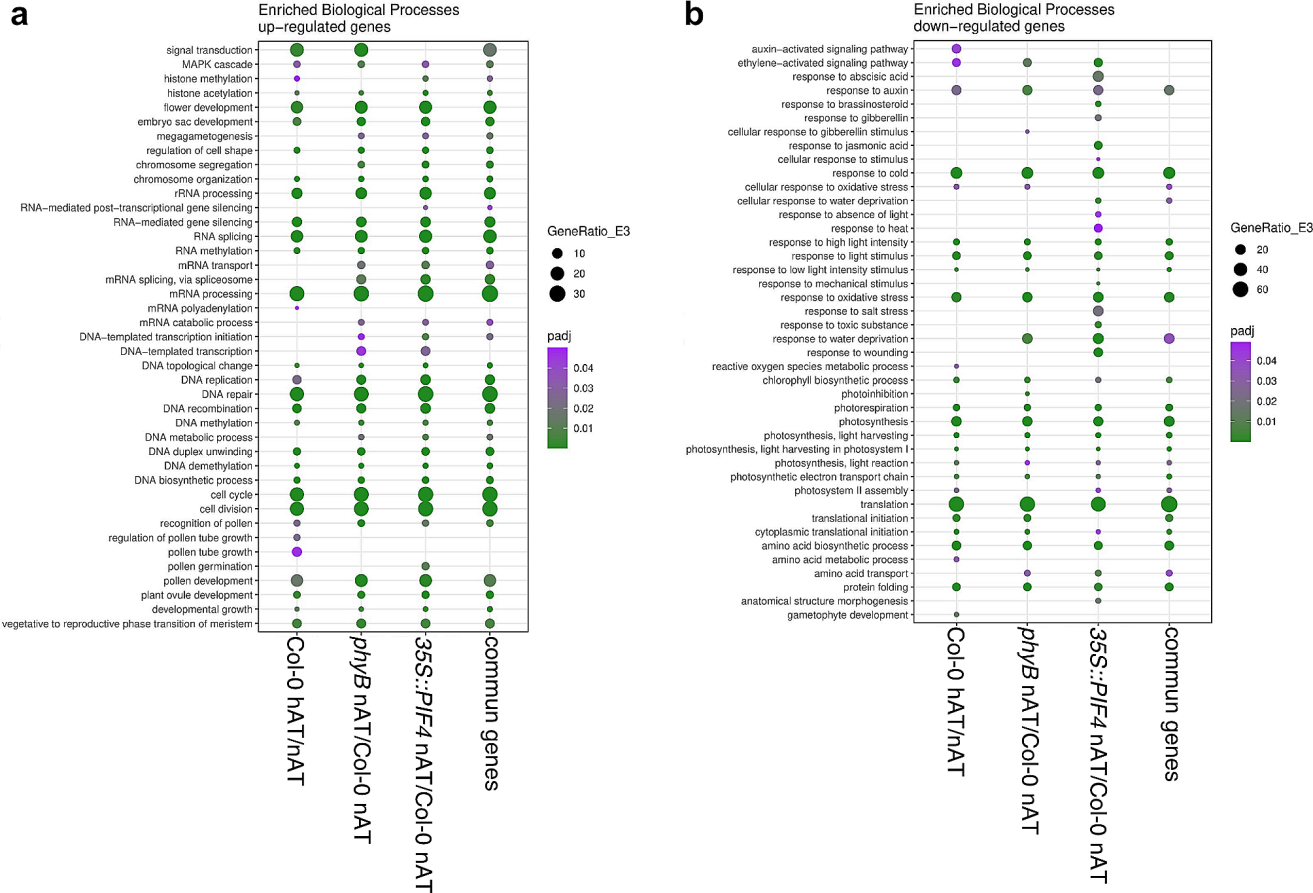
Gene Ontology analysis of the enriched biological processes in pistils of Col-0, *phyB*, *35S::PIF4* and common genes to the three genotypes. Analysis of Gene Ontology (GO) functional annotation of the enriched biological processes was performed for up- (**a**) and downregulated (**b**) DEGs in wild-type pistils from plants grown on hAT, and *35S::PIF4* and *phyB* pistils from plants grown on nAT. Data source is provided in Additional File 4 – Table S2

Gene Ontology (GO) functional annotation analysis was performed for up- and downregulated DEGs in wild-type pistils from plants grown on hAT vs. nAT, and *35S::PIF4* and *phyB* pistils vs. wild-type plants grown on nAT to determine whether the hAT response in wild-type pistils shares GO patterns with the response in pistils from plants defective in the phyB pathway (Fig. 6; Additional File 4 – Table S2).

Cell division rate is known to be dependent on ambient temperature [59]. Several GO terms related to cell division, cell cycle, DNA replication, and mRNA processing were enriched among the commonly upregulated DEGs. These processes are known to be critical during pistil and ovule development. Indeed, GO terms associated with megagametogenesis, ovule, embryo sac, and flower development and the transition to the reproductive phase in the meristem were among the commonly upregulated DEGs (Fig. 6a). Among the GO terms related to fertilization and reproduction, recognition of pollen, (regulation of) pollen growth and pollen development were enriched. Genes involved in pollen tube growth were specifically upregulated in the wild-type pistils at hAT (vs. nAT), whereas genes involved in pollen germination were enriched only in *35S::PIF4* pistils at nAT (compared with nAT wild type) (Fig. 6a). This suggests that both hAT and the phyB-PIF4 pathway may influence the expression of genes involved in ovule development as observed in Fig. 5, and that fertilization processes dependent on pollen tube growth and guidance may be specifically affected by hAT in wild-type pistils.

Surprisingly, GO terms related to the responses to phytohormones and abiotic stresses were found to be downregulated (Fig. 6b). Responses to auxin and ethylene were downregulated in all sample comparisons. However, GO terms associated with brassinosteroids, gibberellins, abscisic acid, and jasmonic acid were exclusively downregulated in *35S::PIF4* pistils at hAT (compared with Col-0 at nAT) (Fig. 6b). Furthermore, GO terms related to cold and light stress responses, photosynthesis, protein translation, and metabolism were generally enriched among the downregulated genes in all three samples (Fig. 6b).

### The expression profile of the *phyB* and *35S::PIF4* pistils at nAT for auxin signaling and miRNA processing genes is comparable to that of wild-type pistils at hAT

**Fig. 7:**
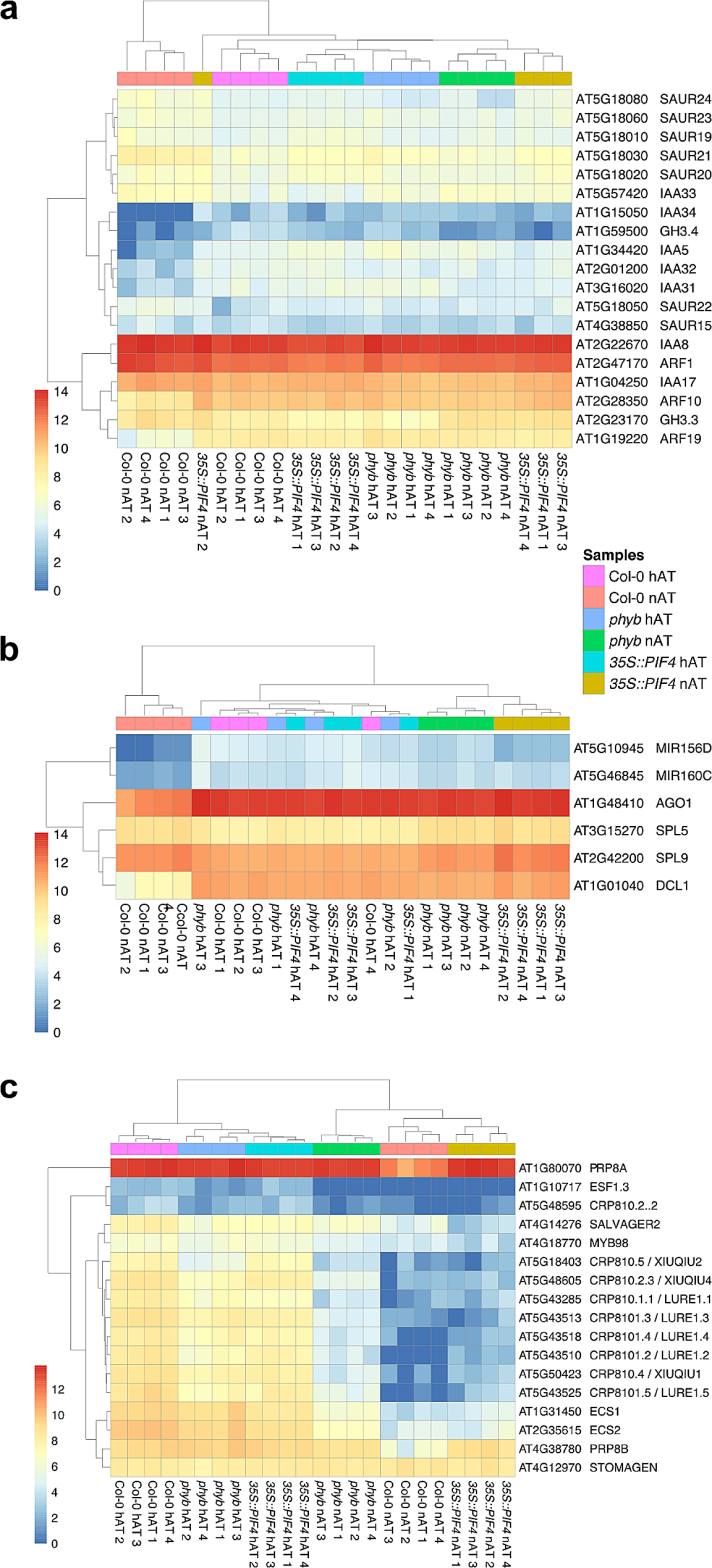
Cluster analysis of DEGs related to auxin (**a**), MIR biogenesis (**b**) and pollen tube attractants (**c**). The color code of the samples is provided. Data source is provided in Additional File 4 – Table S3

Hierarchical clustering analysis of the expressed genes identified two major clusters among the top 100 DEGs in Col-0 pistils at hAT versus nAT (Additional File 1 – FigureS10; Additional File 4 – Table S3), the DEGs involved in the auxin signaling pathway (Fig. 7a; Additional File 4 – Table S3) and in miRNA biogenesis (Fig. 7b; Additional File 4 – Table S3). One cluster consists exclusively of the wild-type pistils from plants grown in nAT. The second cluster includes the pistils from *phyB* and *35S::PIF4* plants grown in nAT and hAT, as well as from wild-type plants grown in hAT. Similar to what was observed during our phenotyping analysis, these results indicate that the response to hAT and to the phyB-PIF4 pathway share a gene regulatory network.

PIF4 binds to the promoters of several *miR156* genes to repress their expression, resulting in the accumulation of the miR156 target transcripts, the *SQUAMOSA-PROMOTER BINDING PROTEIN-LIKE* (*SPL*) genes [60]. SPL will then regulate plant growth in response to shade and warm temperature. The module miR156/*SPL9* regulates the thermomorphogenetic response of the hypocotyl by mitigating its sensitivity of auxin [61]. Several *SMALL AUXIN UP RNA* (*SAUR*) and *Aux/IAA* genes, as well as *AUXIN RESPONSE FACTOR ARF10* and *ARF19* are upregulated in the second cluster (Fig. 7a). We also identified *MIR156*, *MIR160*, and the miRNA processing *AGO1*, *DCL1* genes to be upregulated in the same cluster, while the MIR156 targets *SPL5* and *SPL9* were slightly down-regulated (Fig. 7b).

We looked in more details at the expression behavior of known transcriptional targets of PIF4 (Additional File 4 – Table S4). In the wild-type pistil samples, 3,453 genes are downregulated under hAT compared to nAT. Among these, 522 genes are known targets of PIF4 identified by the TF2Network (http://bioinformatics.psb.ugent.be/webtools/TF2Network/) [62]. 280 of the 522 PIF4 target genes were differentially expressed (downregulated) in the *35S::PIF4* pistils compared to the Col-0 under nAT.

### Pollen tube attractants are upregulated at hAT

We also performed a hierarchical clustering for genes related to pollen tube guidance, an enriched GO term category (Figs. 6a and 7c; Additional File 4 – Table S3). Again, two distinct clusters related to the hAT response (independently of the genotype) were identified. Genes encoding the defensin-like pollen tube attractants CYSTEINE-RICH PEPTIDE (CRP) AtLURE1s and XIUQIU, EMBRYO SURROUNDING FACTORS 1.3 (ESF1.3), EGG CELL SPECIFCs (ECSs), and MYB98, a transcription factor controlling their expression [63, 64], were upregulated in the cluster comprising all pistil samples from plants grown in hAT (Fig. 7c), regardless of genotype.

### Changes in *YUCCA* and *TAA1* expression levels in hAT in mature ovules suggest a role for auxin biosynthesis in the response to high ambient temperature

**Fig. 8:**
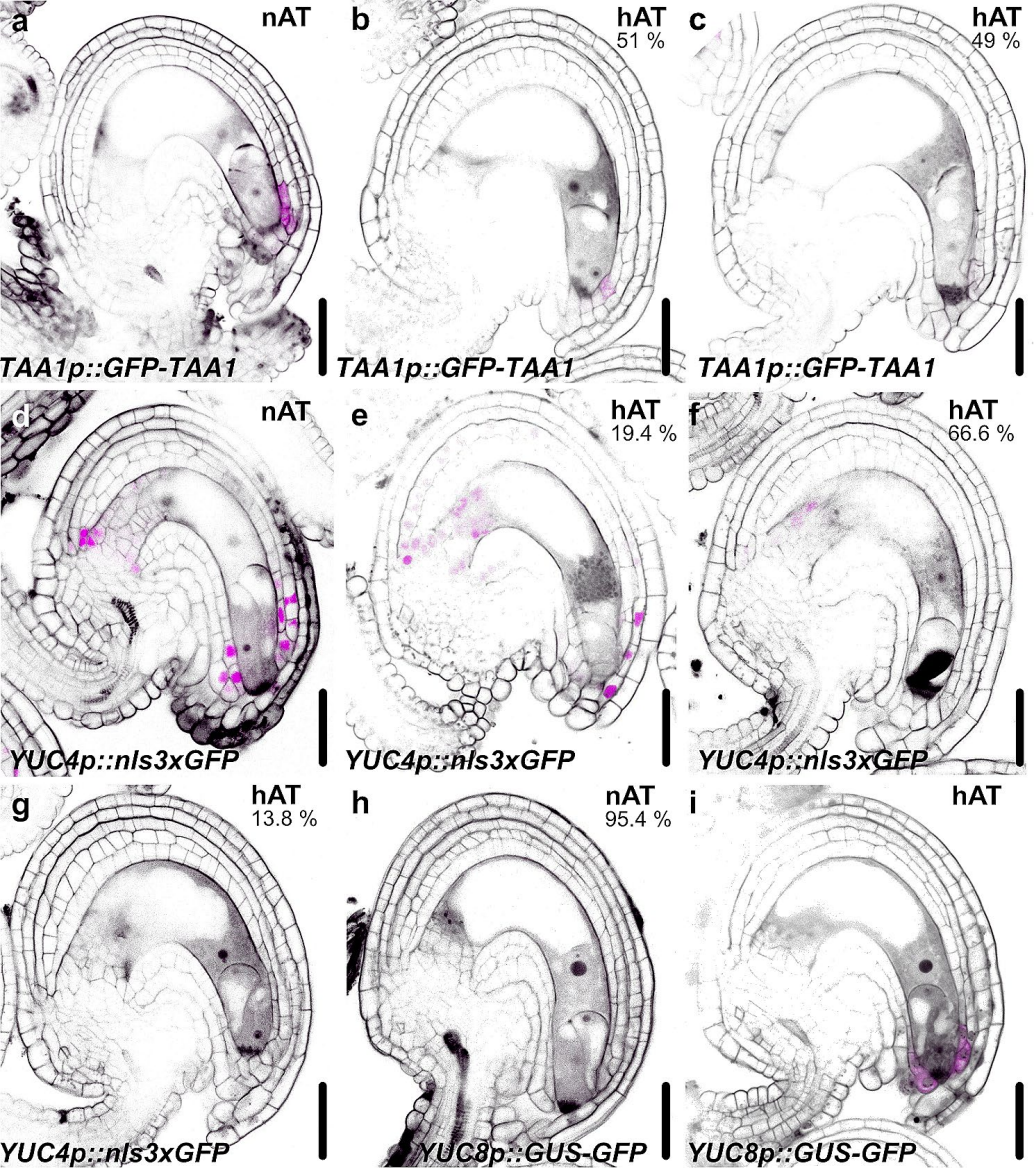
The expression pattern of auxin biosynthetic genes is altered in ovules at hAT. Expression pattern of *TAA1* (**a**-**c**), *YUC4* (**d**-**g**) and *TAA1* (**h**,** i**) in mature ovules from plants grown at nAT (**a**, ** d**,** h**) and hAT (**b**,**c**, **e**, **f**, **g**, **i**). The green fluorescence signal of *TAA1::GFP-TAA1*, *YUC4::nls3xGFP* and *YUC8::GFP-GUS* is seen as magenta, Scale bars represent 20 μm

In seedlings, hAT-activated PIF4 enhances the expression of the *TRYPTOPHAN AMINOTRANSFERASE OF ARABIDOPSIS 1* (*TAA1*), *YUCCA 8* (*YUC8*) and *SAUR* genes in the leaves and hypocotyls [9, 20]. *TAA1*, *YUC4* and *YUC8* are also expressed in mature ovules at the micropyle cells surrounding the embryo sac [65]. To evaluate the effects of hAT on auxin homeostasis in mature ovules, we analyzed the expression pattern of the three auxin biosynthetic genes. *TAA1* is expressed in the micropylar cells in nAT and its expression is altered in hAT (Fig. 8a-c). The *TAA1* fluorescence signal was not detected in 49% of the oules and was weak in the remaining samples in hAT (Fig. 8b and c). *YUC4* was strongly expressed in the integuments of mature nAT ovules (Fig. 8d). Different levels of the fluorescence signal intensity were observed for *YUC4* in hAT ovules: same expression pattern with reduced signal intensity (19.4%; Fig. 8e) restricted expression domain at the chalazal integuments with weak signal intensity (66.6%; Fig. 8f, and no signal (13.8%; Fig. 8g. *YUC8* showed no (95.4%; Fig. 8h to weak expression in the micropylar cells (4.6%) in nAT vules. However, in hAT, *YUC8* was highly expressed in the micropylar cells (Fig. 8i). *YUC8* is known to be upregulated in hAT in other tissues [9], which is consistent with our observations in ovules. The contrasting expression behavior of *YUC4* and *YUC8* at hAT suggests an intricate and complex regulatory mechanism in the response to hAT in the ovules.

### Effects of hAT on early embryo development

Given the effects of hAT on ovules and the transcriptional changes associated with pollen guidance and its impact on fertilization, we investigated the effects of hAT on seed and embryo development in the same genotypes. Seeds bearing embryos from early developmental stages (one-cell to late globular) were analyzed for embryo patterning defects. In nAT, no significant differences were observed between the different genotypes (Table 4). In hAT, however, all genotypes were significantly affected. No statistically significant differences in the percentage of defective embryos were observed between wild type (40.77%), *pif4* (44.23%), *pifq* (41.56%), and *phyB* (30.85%). Only *35S::PIF4* appeared to be resistant to growth at hAT with a significantly lower embryonic defect rate of 21.95% (Table 4). A variety of embryonic defects have been observed, including an excess of cell divisions within the proper embryo or suspensor, irregularities in the size of the hypophysis cell, and a reduction in the length of the suspensor (Fig. 5f-h; Additional File 2 – Table S6). A shorter suspensor was observed in all the genotypes for hAT (Fig. 5h). In nAT, the suspensor of the *35S::PIF4* embryos was longer (111 μm) than the wild-type suspensor (97.18 μm). However, this difference disappeared in hAT, suggesting that the *35S::PIF4* embryos were the most affected by temperature variation for suspensor growth (Fig. 5h; Additional File 2 – Table S6). These results suggest that ectopic overexpression of *PIF4* may confer a minor temperature resistance during embryogenesis.

**Table 4:** Embryonic defects in seeds grown at nAT and hAT

Genotype	Growth conditions	Normal	Defective	*n*	% Defects
Col-0	nAT	121	3	124	2.42
Col-0	hAT	61	42	103	40.77 ***
*phyB*	nAT	111	4	116	3.4
*phyB*	hAT	65	29	94	30.85 ***
*35S::PIF4*	nAT	127	5	132	3.79
*35S::PIF4*	hAT	32	9	41	21.95 *** #
*pif4*	nAT	90	4	96	4.1
*pif4*	hAT	29	23	52	44.23 ***
*pifq*	nAT	145	8	153	5.23
*pifq*	hAT	45	32	77	41.56 ***

Fisher’s Exact Test was used for statistical analysis of these comparisons. To ensure result reliability, anthers from each genotype and condition were examined across three replicates. Significance indicators include * for a significant temperature effect and # for a significant genotype effect. P-values are denoted as # (0.05 − 0.01) and *** (< 0.00009)

### hAT-induced changes in seed traits

**Fig. 9:**
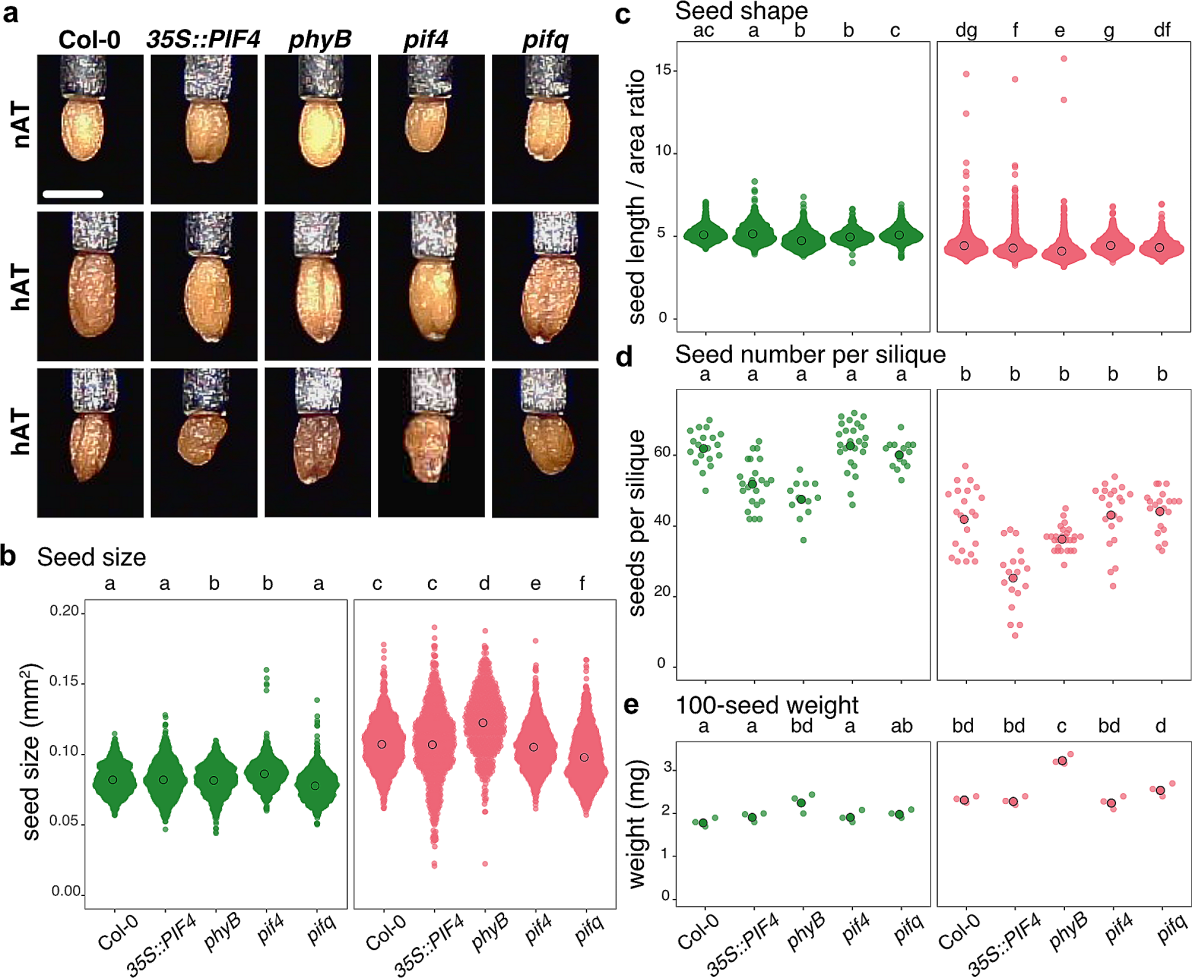
Temperature exposure leads to fewer, larger, and rounder seeds. (**a**) Dry seed phenotype from Col-0, *35S::PIF4*, *phyB*,* pif4*, and *pifq* plants grown at nAT and hAT. Two representative pictures of seed phenotypes are ahown. The scale bar represents 0.5 mm. (**b**, **c**) Seed size in mm^2^ (**b**) and seed shape (length to seed area ratio) (**c**) evaluations. The surface area and shape (seed length/ seed aera) of 1 000 seeds for each genotype were analyzed in triplicate from plants grown at nAT and hAT. (**d**) The number of seeds produced per silique is calculated in at least 12 mature siliques for each genotype/condition in triplicates. (**e**) The weight of 100 seeds from each genotype is measured in triplicates. Genotypes that share the same letter are not statistically significantly different. Data source are provided in Additional File 3 – Tables S8 and S9

Dry seeds harvested from the same plants flowering at nAT and hAT were phenotyped using the Boxeed robot. We focused on four seed traits: number of seeds produced per silique, seed shape, seed size, and seed weight (Fig. 9; Additional File 3 – Tables S8 and S9). Elevated ambient temperatures led to an increase in seed area in all the genotypes, with the production of larger viable seeds and smaller misshapen seeds (Fig. 9a). Seed area increased by 34.74% in Col-0, 31.73% in *35S::PIF4*, 47.83% in *phyB*, 25.20% in *pif4*, and 47.67% in *pifq* (Fig. 9b; Additional File 2 – Table S7). Additionally, seeds produced under hAT were rounder across various genotypes, as assessed by the ratio of the seed length to the seed area. The *phyB* seeds were the most affected by shape changes in hAT (Fig. 9c; Additional File 2 – Table S8). Evaluation of the number of seeds per silique showed that all genotypes produced fewer but heavier seeds per silique at hAT in all the genotypes (Fig. 9d and e; Additional File 2 – Tables S9 and S10). Interestingly, at nAT, *phyB* seeds were by 25% heavier than wild-type seeds (Fig. 9e; Additional File 2 – Table S10). The higher seed weight observed in seeds developed at hAT suggests a possible adaptive strategy in which plants may favor the production of nutrient-rich seeds rather than a greater number of seeds. However, *phyB* plants grown on nAT and wild-type plants grown on hAT produced a comparable number of seeds, precisely 42.14 and 47.75 seeds per silique for a comparable weight, 2.33 mg and 2.26 mg per 100 seeds, respectively.

### The correlation of the hAT response in reproductive tissues

A correlative analysis of the effects of hAT on reproduction in Col-0 plants (Fig. 4b) showed that seed number and the increased number of embryo defects and pollen defects were significantly negatively correlated (-0.92 and − 0.63, respectively). Seed number and seed weight were also significantly negatively correlated (-0.71). Surprisingly, pollen defects were positively correlated (0.87) with increased seed weight.

## Discussion

Plants have adapted to ambient growth temperatures through various molecular mechanisms, including the phyB-PIF4 pathway [13, 66]. While the response of *Arabidopsis thaliana* seedlings to high ambient temperatures has been studied previously, we focused our study on other processes: adult traits and reproductive growth. We also asked whether the phyB-PIF4 pathway may also be involved in those responses. Therefore, we performed a comprehensive morphological analysis of different organs during both vegetative and reproductive growth stages using automated phenotyping solutions, with the *phyB* mutant and *PIF4* overexpression lines. We uncovered how repression of the phyB-PIF4 pathway differentially and pervasively induces thermomorphogenesis, thereby affecting the plant’s adaptation to suboptimal temperatures. The phenotypic analysis was complemented by the study of the transcriptional changes in pistils to help overcome the hAT-reduced fertilization rate.

Research on the impact of hAT on root system growth has yielded mixed results, with some studies reporting decreased root growth and others reporting increased root growth [67–70]. In our study, hAT enhances root elongation in all genotypes, although the impact did not reach statistical significance, possibly due to sample size or resolution limitations. hAT prompts roots to prioritize elongation over lateral root development, resulting in a less dense but elongated root system. Adventitious root, which, like lateral roots, emerge post-embryonically, serves as a crucial plant strategy to cope with environmental stresses [71]. We found that hAT induced adventitious root formation in all studied lines, except when *PIF4* expression was altered. Overexpression of *PIF4* and repression of phyB disrupt growth rates under nAT, opposite to the impact of hAT. Additional mechanisms likely contribute to root responses to hAT, underscoring the complexity of the responses [50]. This is consistent with previous findings showing that overexpression of *PIF4* hinders the thermal response of roots, similar to the phenotypes of *hy5* and *phyA phyB* mutants. Reduction of root meristem size in hAT is dependent on phyA and phyB [72, 73]. With a different temperature settings, Song et al. (2017) observed that a short-term heat shock at 37 °C inhibited primary root elongation in wild type and, more intensively, *phyB* and *phyA* mutants [67]. Despite differing temperature conditions leading to contrasting effects on lateral root growth observed in their study compared to ours, *phyB* mutants consistently resisted the temperature-induced response in both investigations.

Interestingly, our results highlight the divergent response of shoot and root development to high temperatures. While hAT inhibits shoot elongation, it does not affect the final root length. However, when plants are exposed to hAT, initial growth acceleration and reduced branching are common in both tissues. It seems that both the root and shoot prioritize initial growth at hAT, likely as a strategy to distance themselves from the warm soil surface. This prioritized growth, particularly evident in the root, comes at the expense of nutrient uptake, as indicated by the observed reduction in the number of emerged lateral roots. This trade-off underscores the dynamic adjustments that plants make in response to environmental stress and highlights the intricate balance between growth and resource allocation. Notably, *PIF4* overexpression abolishes the temperature response of both root and shoot branching, suggesting a potential function of this transcription factor in shoot and root development at hAT.

Flowering time in plants is regulated by environmental signals that affect gene expression in the shoot apical meristem. Notably, ambient temperature modulates the expression of *FLOWERING LOCUS T (FT)* [74]. hAT generally leads to earlier flowering responses in most plants (reviewed by [75]). PIF4 emerges as a pivotal player in temperature-induced early flowering in *Arabidopsis*, exerting its influence by binding to the *FT* promoter in a temperature-dependent manner [66]. We have shown that exposing plants to hAT results in the premature cessation of rosette growth, leading to a reduced rosette area (Fig. 2). These plants appear to prioritize energy conservation for the reproductive phase, which ultimately means reduced branching. Initially, plants hastened shoot elongation to distance flower buds from the warm soil surface, resulting in earlier flowering (Fig. 3). Most of the temperature effects were observed in the *phyB* mutant line under normal conditions, suggesting that the repression of phyB simulates the effects of hAT during shoot development. This is to be expected as, at hAT, phyB undergoes thermal reversion into the inactive phyB-Pr [12, 18], similarly in the mutated phyB protein in the *phyB* mutant. Furthermore, in agreement with [76], our investigation showed that the studied spectral vegetation indices exhibited increased responsiveness to hAT during later stages of development. This suggests their potential utility as reliable non-destructive indicators of temperature stress.

Plant reproductive development, especially pollen, is highly sensitive to environmental stress [77, 78]. Growing *Arabidopsis* at 27 °C affects pollen development, resulting in male sterility with a 22% reducion in pollen viability, through processes such as meiosis disruption, premature development, and altered hormone regulation [79, 80]. We observed a mild effect of hAT on pollen viability with *pif4* and *pifq* plants being resistant to hAT for the production of viable pollen grains. Our observations revealed a robust phenotypic response to hAT in ovules, highlighting their sensitivity to temperature changes. We demonstrated that the *35S::PIF4* plants in nAT effectively mimic the effects of hAT, highlighting the critical role of this pathway in thermomorphogenesis in female reproductive organs. To investigate the molecular mechanisms involved, we performed transcriptome analyses of wild-type, *35S::PIF4*, and *phyB* pistils from plants grown at nAT and hAT. This comprehensive approach allowed us to compare the transcriptomic responses of these genotypes in response to hAT and understand how the repression of the phyB pathway mimics the expression profile and phenotypes of wild-type pistils exposed to hAT. DEG analysis revealed that wild-type plants show significant up- and downregulation in response to hAT, while this response is milder in *phyB* and *35S::PIF4* pistils. The DEG profiles of *phyB* and *35S::PIF4* at nAT were similar to the response to hAT in the wild type.

We identified that hAT influenced the expression of specific *microRNAs*, particularly *MIR156. MIR156* has been implicated in *Arabidopsis* hypocotyl elongation in response to hAT and is upregulated in our transcriptomic data [60, 61]. Consistently, heat stress during cotton pollen development regulates the expression of 6,281 genes, among which *miR167* and *miR396* are associated with pollen fertility by targeting genes involved in auxin signaling and metabolism pathways. Additionally, heat-induced jasmonic acid (JA) signaling activates genes associated with auxin synthesis, ultimately leading to pollen abortion [81]. Furthermore, *miR167* downregulates the expression of *ARF6* and *ARF8* genes in *Arabidopsis* ovules, facilitating integument growth. In anthers, *miR167* affects gene expression in connective cells and locules, thereby influencing pollen release. The regulatory function of *miR167* underscores its essential role in patterning during the development of reproductive organs [82]. These findings suggest that miRNAs play crucial roles in reproduction and response to hAT, potentially acting as mediators linking high-temperature signaling pathways to hormone signaling pathways during reproductive organ development.

The impact of hAT on plant reproductive development involves complex regulatory mechanisms. While elevated temperature has been reported to activate auxin biosynthesis in vegetative plant tissues, such as the hypocotyl, it has opposite effects on auxin levels and biosynthetic genes during anther development in barley and *Arabidopsis*. Specifically, elevated temperature repressed the expression of *YUCCA* auxin biosynthetic genes, resulting in reduced endogenous auxin levels in developing anthers [83–85]. Similarly, our transcriptome analysis reveals that at hAT, auxin biosynthetic genes are downregulated at hAT during ovule development, which we confirmed using fluorescent reporters (Fig. 8). Furthermore, Gene Ontology terms associated with the “auxin-activated signaling pathway” and “response to auxin” are suppressed at hAT.

Despite a more pronounced impact on male processes, it is important to note that female tissues and post-fertilization development are also highly sensitive to temperature variation (reviewed by [86]). Elevated temperatures significantly influence seed production and overall plant yield. Despite extensive research on temperature effects on pollen and seed development, the underlying molecular mechanisms remain unclear. hAT affects both the total number of ovule/seeds and the number of mature ovule/seeds per pistil/silique. Synergid cells produce and secrete defensin-like proteins as pollen tube attractants to guide the pollen tube to the embryo sac for an effective fertilization [63]. This mechanism ensures successful seed production. GO terms related to fertilization and pollen tube growth and guidance were enriched in hAT samples. The genes encoding defensin-like pollen tube attractants were upregulated at hAT samples, independently of the genotypes. Contrastingly, an increased number of defective ovules, including synergid collapsed, were observed at hAT in all genotypes, affecting fertilization rate and seed set. The connection between the increased expression of the pollen tube attractants, the ovule phenotype, and decreased fertilization rate is unclear and will require further investigation. In the *Arabidopsis* Burren ecotype, warm temperatures resulted in up to 43% unferilized ovules, leading to shorter siliques and reduced seed yield while promoting larger seeds [87]. A 7 °C increase in temperature (reaching 30 °C) negatively affects multiple reproductive traits in *Arabidopsis*, including fewer ovules per pistil, fewer anthers and pollen grains per flower, and an increased incidence of improperly developed ovules leading to abortion [88]. In our study, hAT affected sexual reproductive organs and seed-related processes, influencing overall seed yield. Phenotyping with Boxeed identified larger and heavier seeds in hAT, possibly compensating for the reduced seed set (Fig. 9). Repressing phyB enhanced PIF4 activation, heightening plant sensitivity to elevated temperatures during both male and female reproduction. Surprisingly, this mechanism improves plant resistance to hAT during embryogenesis, suggesting a versatile molecular pathway across developmental stages.

## Conclusions

Our study provides an in-depth look at the plant thermomorphogenesis response during their vegetative and reproductive stages through a comprehensive combination of automated phenotyping approaches and image analysis. We found that high ambient temperatures alter the timing of events like flowering and affect basic growth patterns, such as shoot and root system architecture. This suggests that plants prioritize reproduction under challenging conditions, a shift underscored by different temperature sensitivities at different developmental stages. Key among our findings is the role of the phyB-PIF4 pathway, especially in regulating the development of reproductive tissues. However, its influence is less pronounced during embryogenesis. Overall, our research highlights the complex interplay between plant development and environmental temperatures, with the phyB-PIF4 pathway playing a significant role in plant thermomorphogenesis.

### Electronic supplementary material

Below is the link to the electronic supplementary material.

Additional File 1

Additional File 2

Additional File 3: Table S1

Additional File 3: Table S2

Additional File 3: Table S3

Additional File 3: Table S4

Additional File 3: Table S5

Additional File 3: Table S6

Additional File 3: Table S7

Additional File 3: Table S8

Additional File 3: Table S9

Additional File 4: Table S1

Additional File 4: Table S2

Additional File 4: Table S3

Additional File 4: Table S4

## Data Availability

The dataset supporting the conclusions of this article is deposited to the NCBI repository (BioProject accession number PRJNA1091589, https://www.ncbi.nlm.nih.gov/bioproject/PRJNA1091589).

## Footnotes

1 Kolde, Raivo. “Pheatmap: pretty heatmaps.” *R package version* 1.2 (2019): 726. https://CRAN.R-project.org/package=pheatmap.

2 Kassambara, Alboukadel. “ggpubr:“ggplot2” based publication ready plots.” R package version 0.1 7 (2018). https://cran.r-project.org/package=ggpubr.

## Abbreviations

ARF: Auxin response factor
CRP: Cystein-rich peptide
DAFD: Days after the development of the first flower
DEG: Differentially expressed genes
ECS: EGG Cell specific
ESF: Embryo surrounding factor
GO: Gene ontology
hAT: High ambient temperature
MCARI1: Modified chlorophyll absorption ratio index 1
nAT: Normal ambient temperature
NDVI: Normalized difference vegetation index
NPQ: Non-photochemical quenching
OSAVI: Optimized soil-adjusted vegetation index
phyB: Phytochrome B
PIF: Phytochrome-interacting factors
PRI: Photochemical reflectance index
PSII: Photosystem II
PSRI: Plant senescence reflectance index
qP: Photochemical quenching coefficient
SAUR: Small auxin up RNA
SIPI: Structure insensitive pigment index
SPL: Squamosa-promoter binding protein-like
TAA1: Tryptophan aminotransferase of Arabidopsis 1
VNIR: Visible and near infrared
YUC: Yucca
