# Supplementary material for: Integrative phenotyping analyses reveal the relevance of the phyB-PIF4 pathway in Arabidopsis thaliana reproductive organs at high ambient temperature
Source: BMC Plant Biol. 2024 Jul 29;24:721. doi: 10.1186/s12870-024-05394-w (PMC11285529; doi:10.1186/s12870-024-05394-w)
Supplement: Supplementary file 1 — Additional File 1 [file 12870_2024_5394_MOESM1_ESM.pdf]

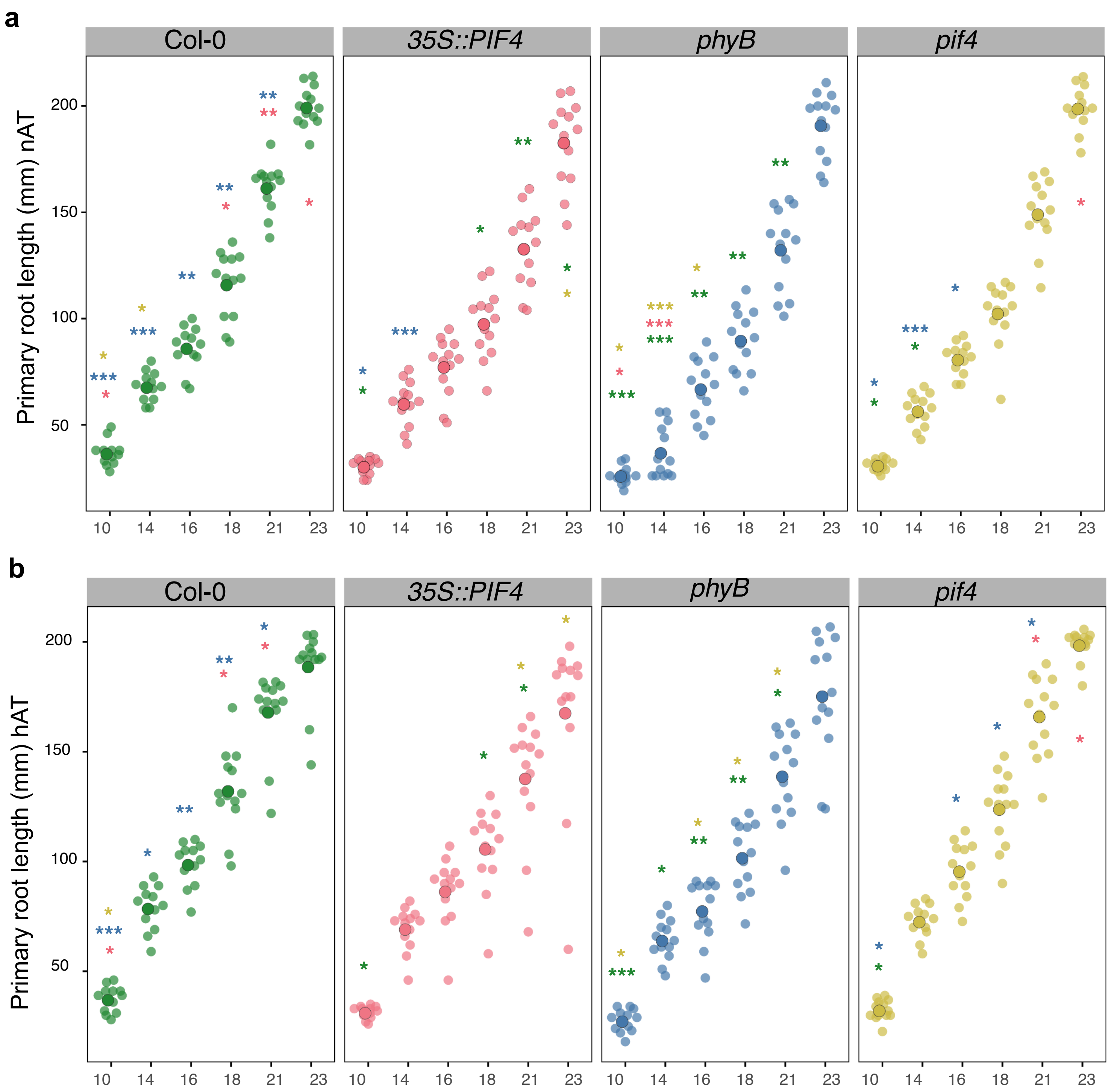

Additional File 1 – Figure S1. Primary root length per genotype
Time series of primary root length per genotypes for seedlings grown in nAT (**a**) and hAT (**b**). Days after sowing are indicated in the x-axis. The root length is indicated as mm. n = 13 plants per genotype. Data source is provided in Additional File 3 – Tables S1 and S2. The significance effects of the genotype are depicted with as *. The *p*-value ranges are specified as * for *p*-values between 0.05 and 0.01, ** for *p*-values between <0.01 and 0.0001, *** for *p*-values lower than 0.0001. The color of the * matches the color of the tested genotype.

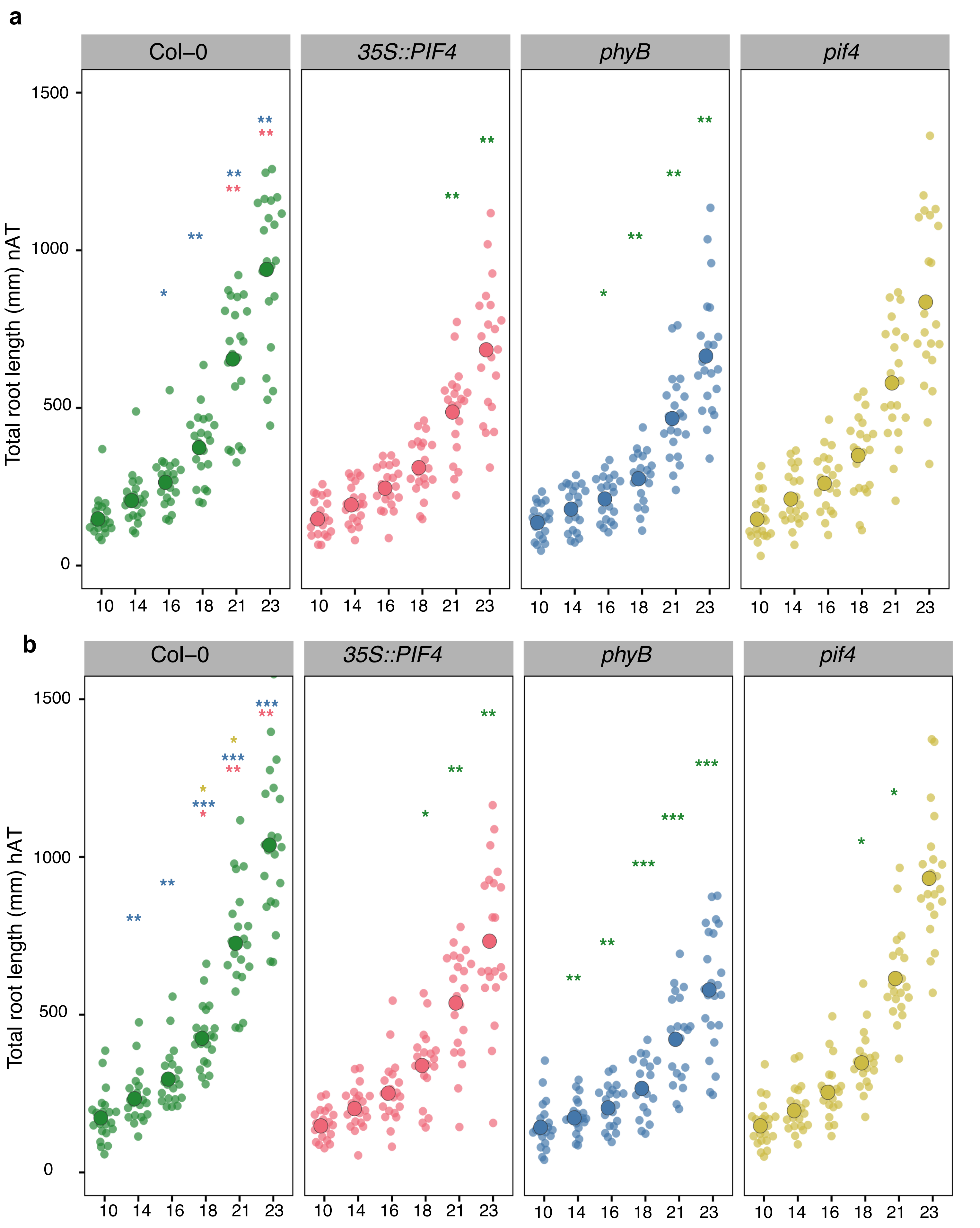

Additional File 1 - Figure S2. Total root length per genotype

Time series of the total root length per genotypes for seedlings grown in nAT (**a**) and hAT (**b**). Days after sowing are indicated in the x-axis. The root length is indicated as mm. n = 13 plants per genotype. Data source is provided in Additional File 3 – Table S2. The significance effects of the genotype are depicted with as *. The *p*-value ranges are specified as * for *p*-values between 0.05 and 0.01, ** for *p*-values between <0.01 and 0.0001, *** for *p*-values lower than 0.0001. The color of the * matches the color of the tested genotype.

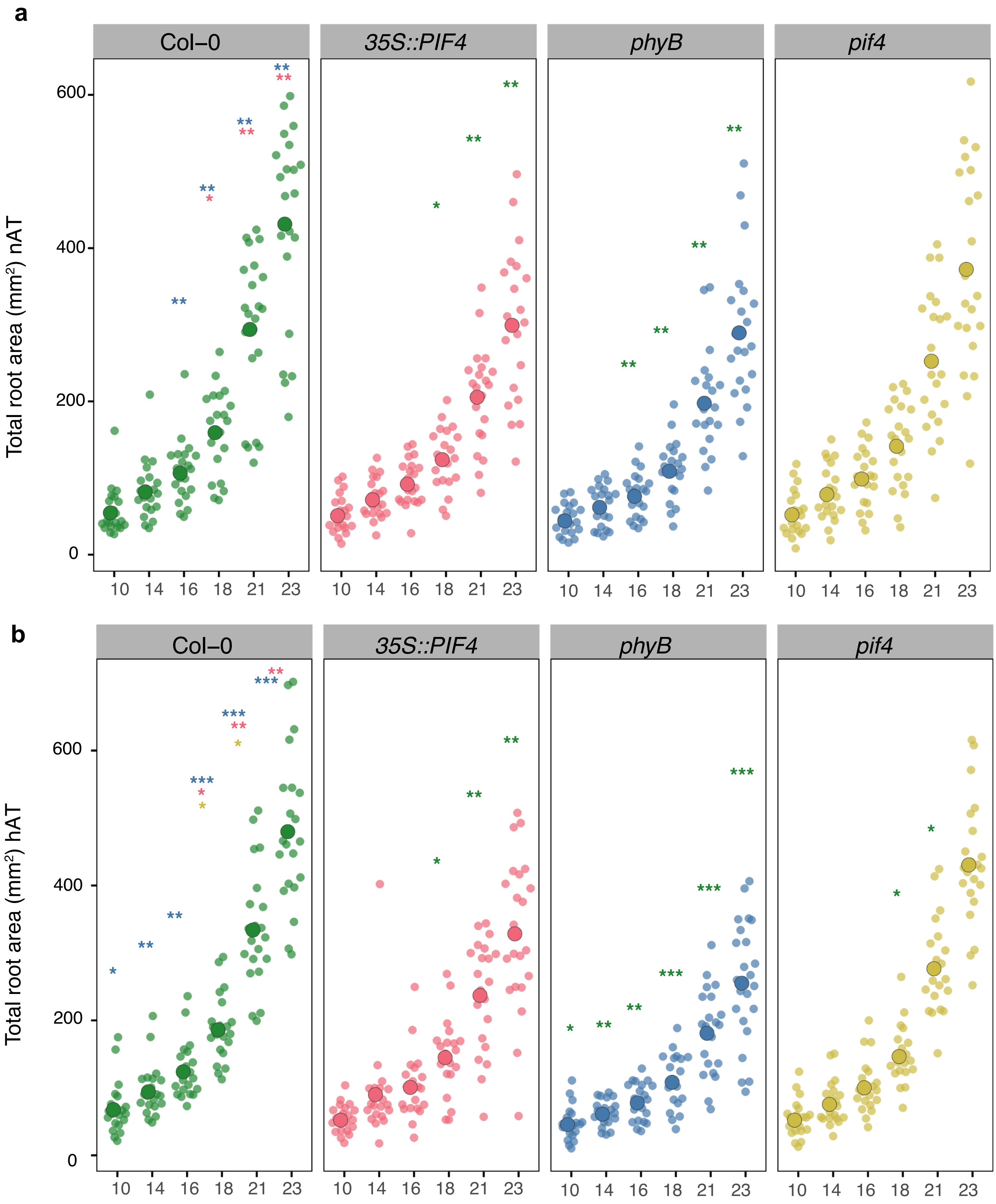

Additional File 1 - Figure S3. Total root area per genotype

Time series of the total root area per genotypes for seedlings grown in nAT (**a**) and hAT (**b**). Days after sowing are indicated in the x-axis. The root length is indicated as mm. n = 13 plants per genotype. Data source is provided in Additional File 3 – Tables S1 and S2. The significance effects of the genotype are depicted with as *. The *p*-value ranges are specified as * for *p*-values between 0.05 and 0.01, ** for *p*-values between <0.01 and 0.0001, *** for *p*-values lower than 0.0001. The color of the * matches the color of the tested genotype.

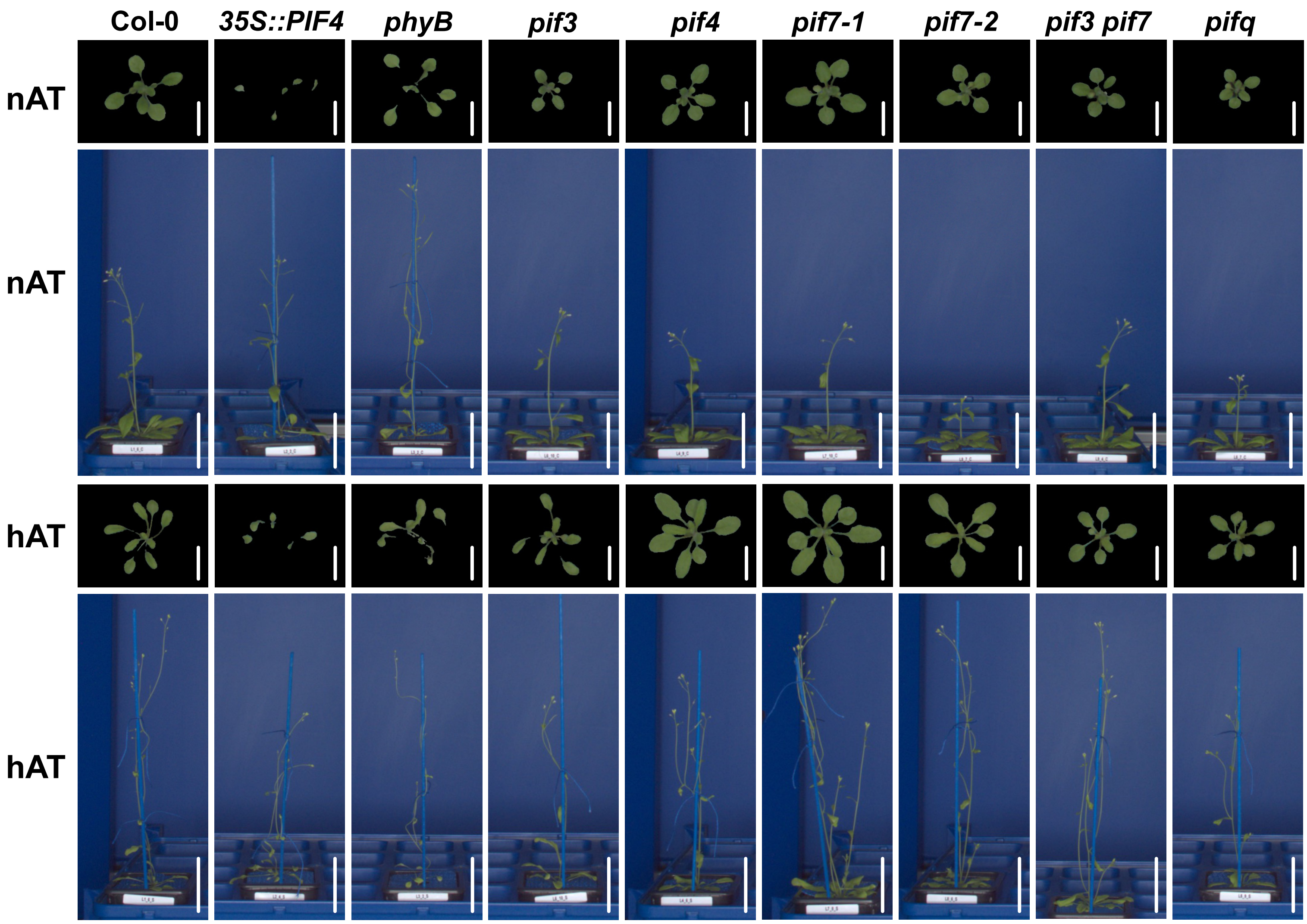

Additional file 1. Figure S4. Plant photos
Photographs of seedlings and plants from Col-0, *35S::PIF4, phyB, pif3, pif4, pif7-1, pif7-2, pif3 pif7,* and *pifq* grown at nAT and hAT. Plants were photographed at 20 days after sowing (top view) and 32 days after sowing for nAT and30 days after sowing for hAT (side view). Scale bars are 2 cm for top views and 5 cm for side view.

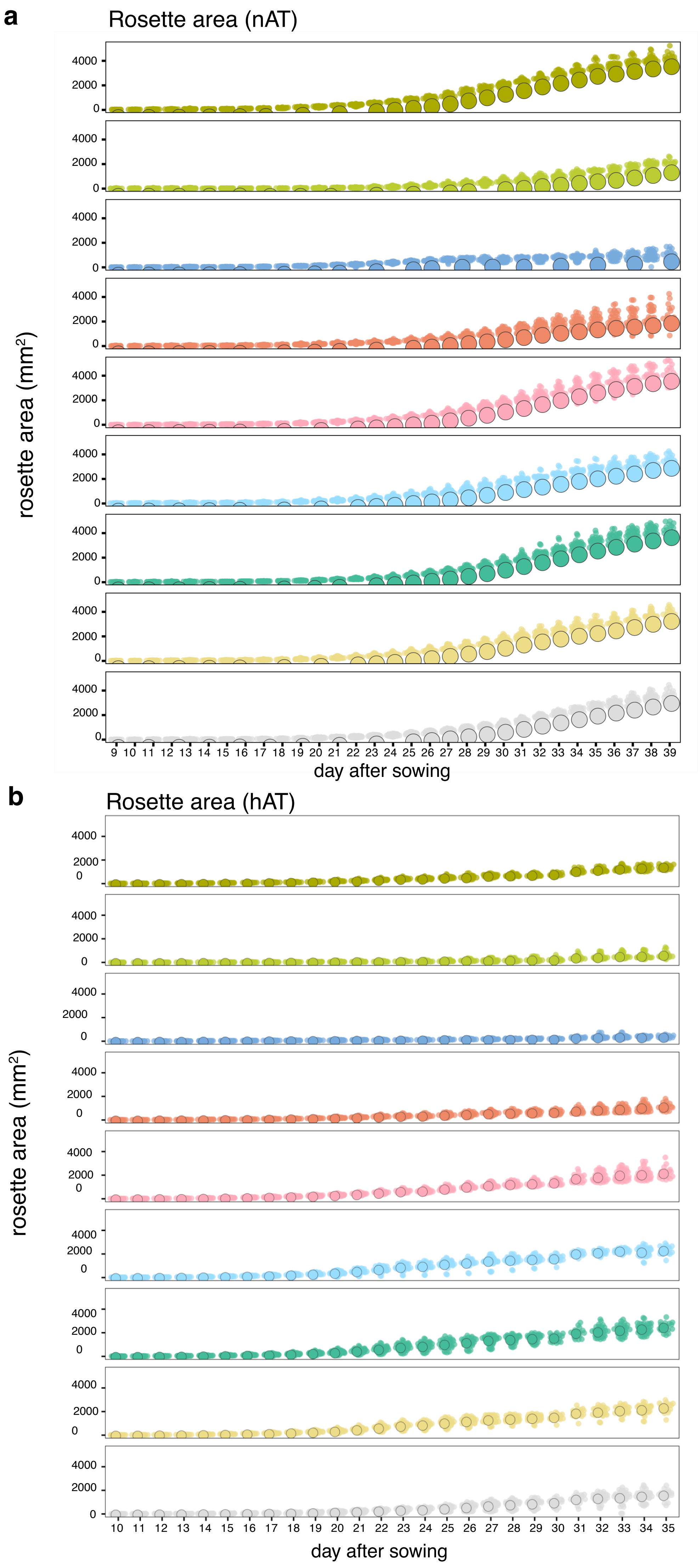

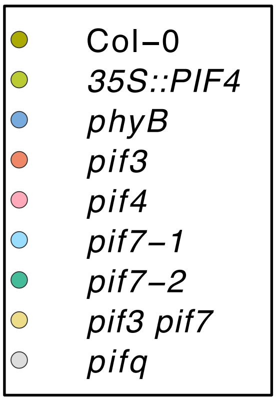

Additional File 1 - Figure S5. Rosette area per genotype. Relates to Figure 2. Time series of the rosette area per genotypes for seedlings grown in nAT (**a**) and hAT (**b**). Days after sowing are indicated in the x-axis. Data source is provided in Additional File 3 – Tables S3 and S4.

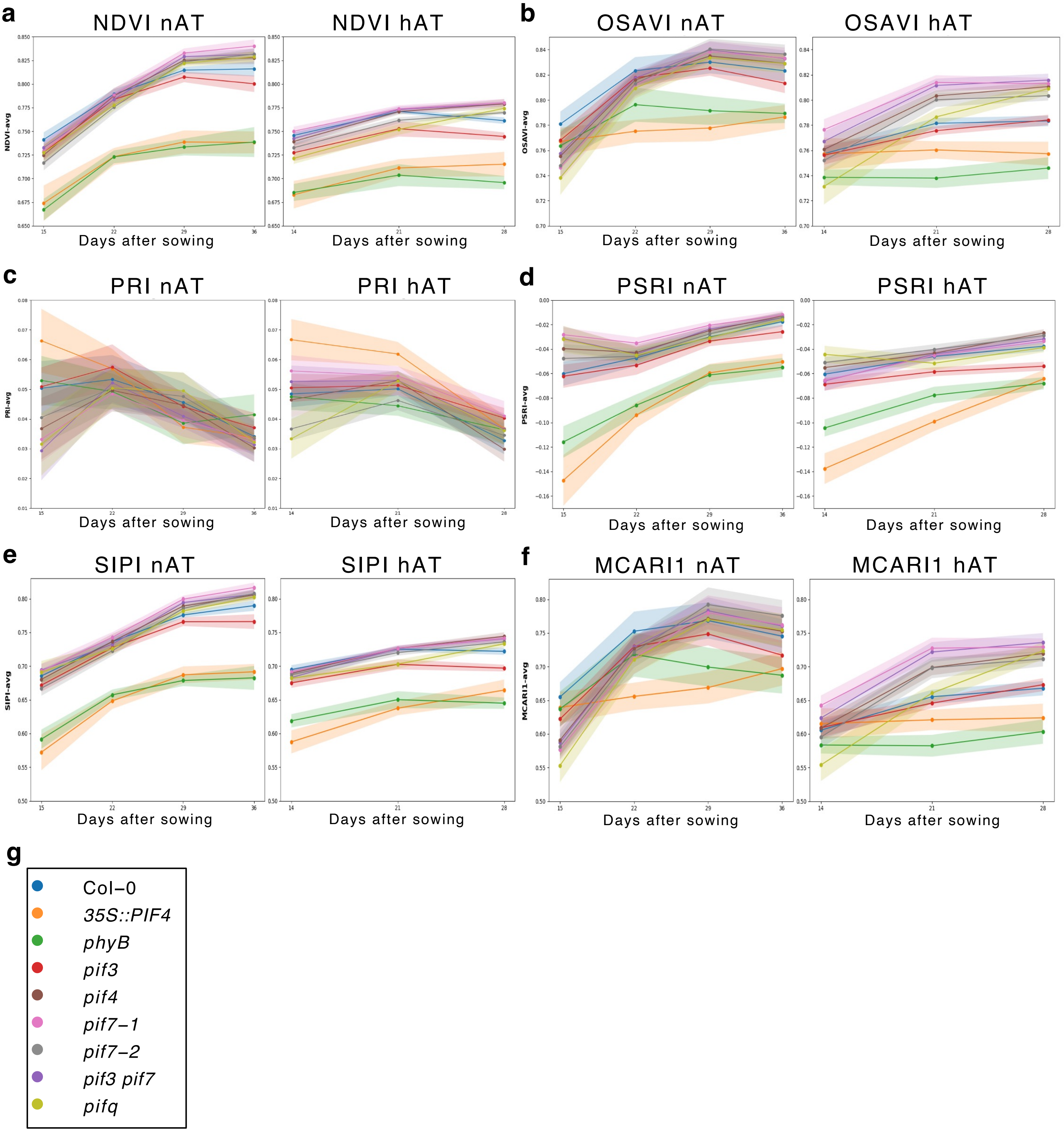

Additional File 1 - Figure S6. VNIR parameters

Graphs depicting the averaged measurements of (**a**) NDVI-avg, (**b**) OSAVI, (**c**) PRI, (**d**) PSRI, (**e**) MCARI1, and (**f**) SIPI, ranging from 15 days to 36 after sowing for nAT (left) and 14 to 28 after sowing for hAT (right). (**g**) The color legend for the genotypes is the same for all the graphs. The averaged values with a 95% confidence interval (shadow) are depicted. Data source are provided in Additional File 3 - Table S6.

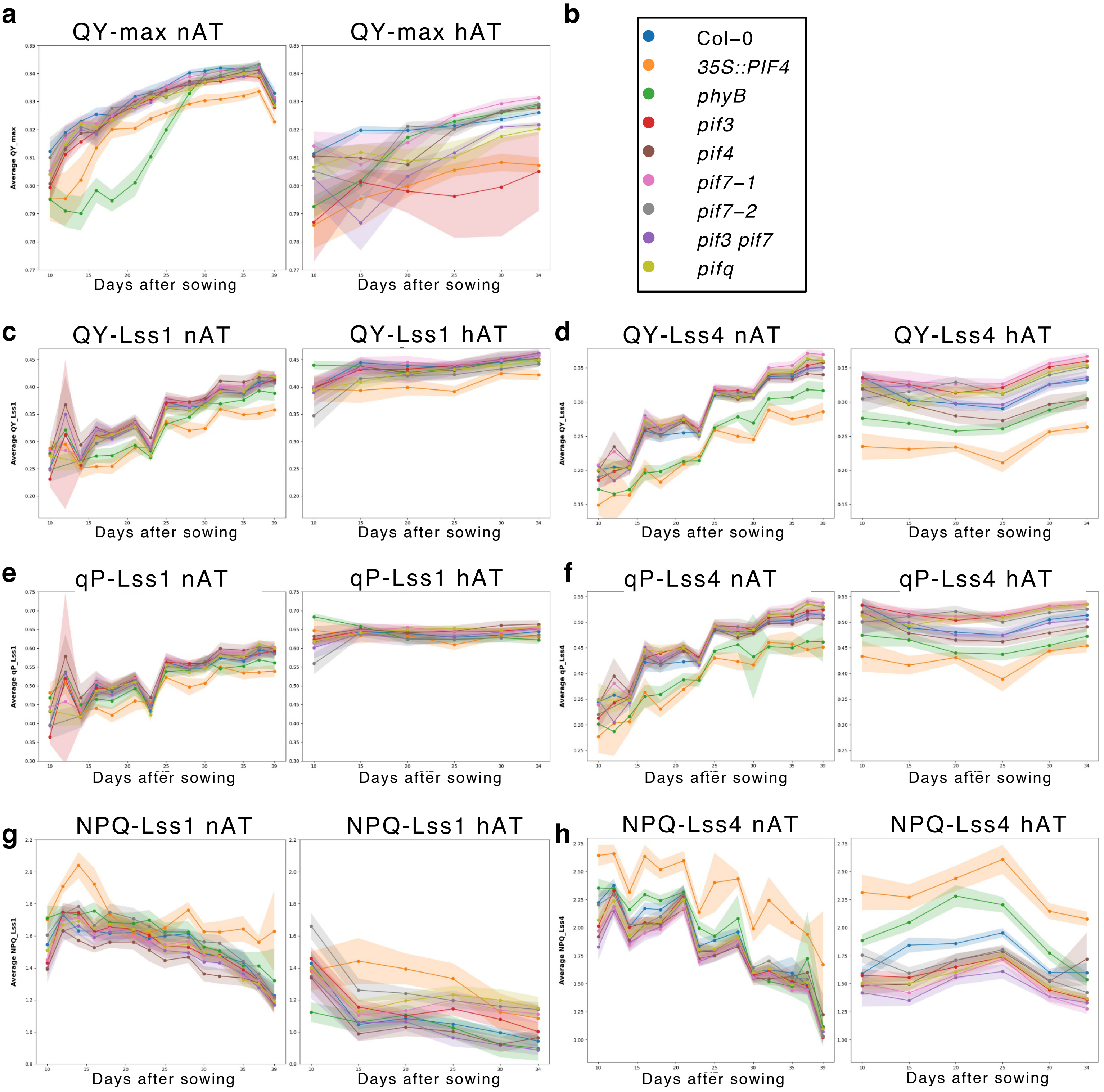

Additional File 1 - Figure S7. Photosynthetic performance

Measurements of QY-max (**a**), QY-Lss1 (**c**), QY-Lss4 (**d**), qP-Lss1 (**e**), qP-Lss4 (**f**), NPQ-Lss1 (**g**), and NPQ-Lss4 (**h**) at nAT (left) and hAT (right), ranging from 10 days to 39 days after sowing for nAT and up to 32 days for hAT. (**b**) The color legend for the genotypes is the same for all the graphs. The averaged values with a 95% confidence interval (shadow) are depicted. Data source are provided in Additional File 3 - Table S7.

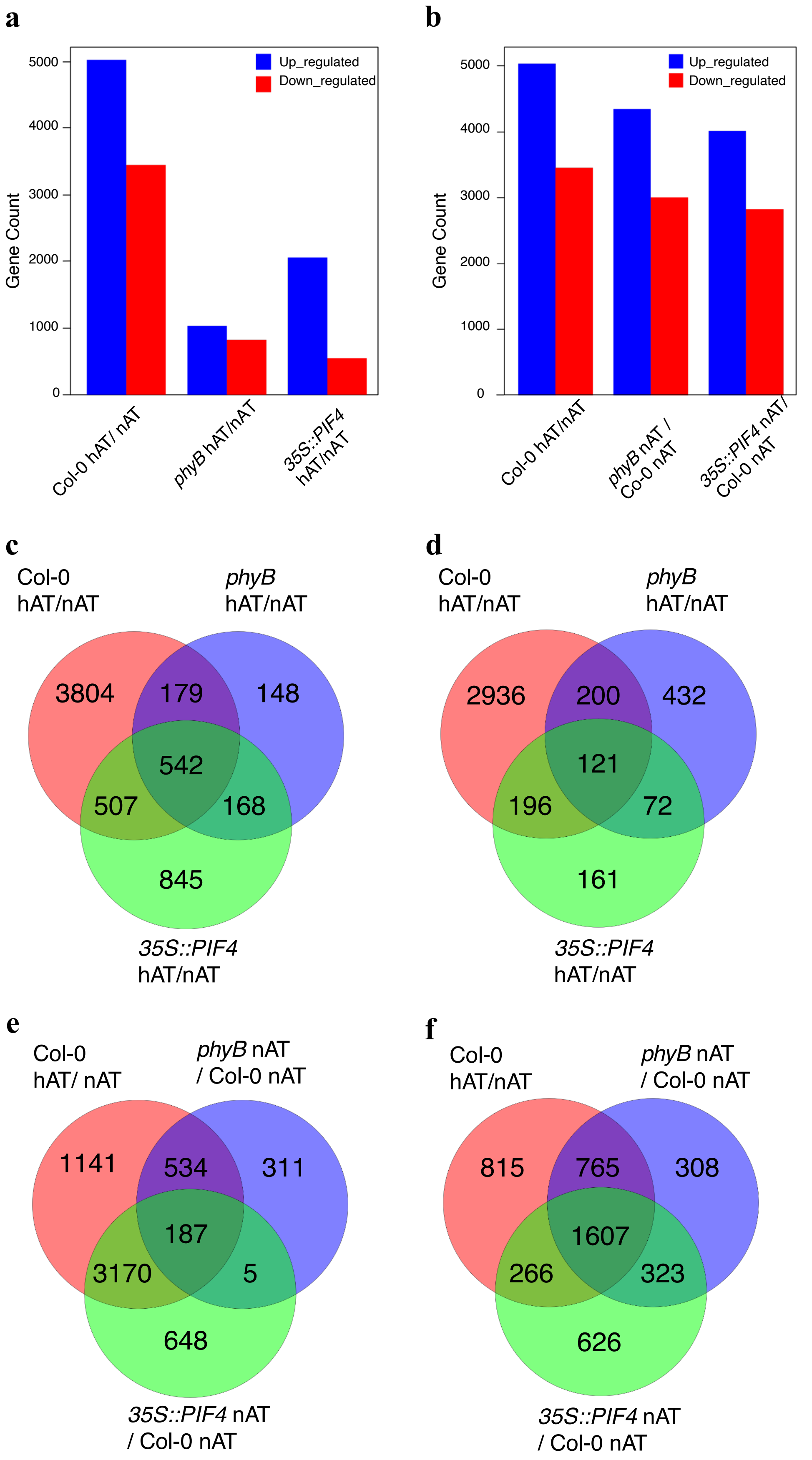

Additional File 1 - Figure S8. DEG and VENN diagrams of the RNAseq experiment.
(**a**) Number of up- and downregulated genes in each genotype, comparing hAT to nAT. (**b**) Number of up- and downregulated genes in Col-0 at hAT and *phyB* and *35S::PIF4* at nAT, normalised to genes expressed in Col-0 pistils at nAT. (**c-f**) Venn diagrams comparing the upregulated (**c, e**) and downregulated (**d, f**) genes in each genotype comparing hAT to nAT (**c, d**) and comparing the response to hAT in Col-0 to the response in *phyB* and *35S::PIF4* pistils at nAT.

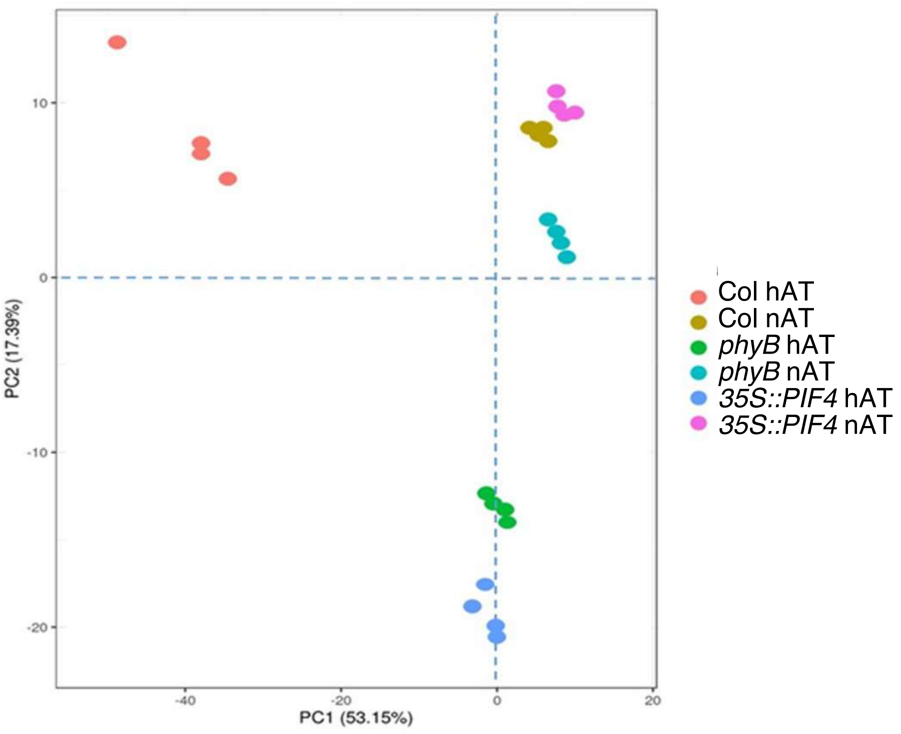

Additional File 1 - Figure S9. Principal Component analysis

Comparing the transcriptomic behaviour of the pistils from Col-0, *phyB* and *35S::PIF4* plants grown at hAT and nAT.

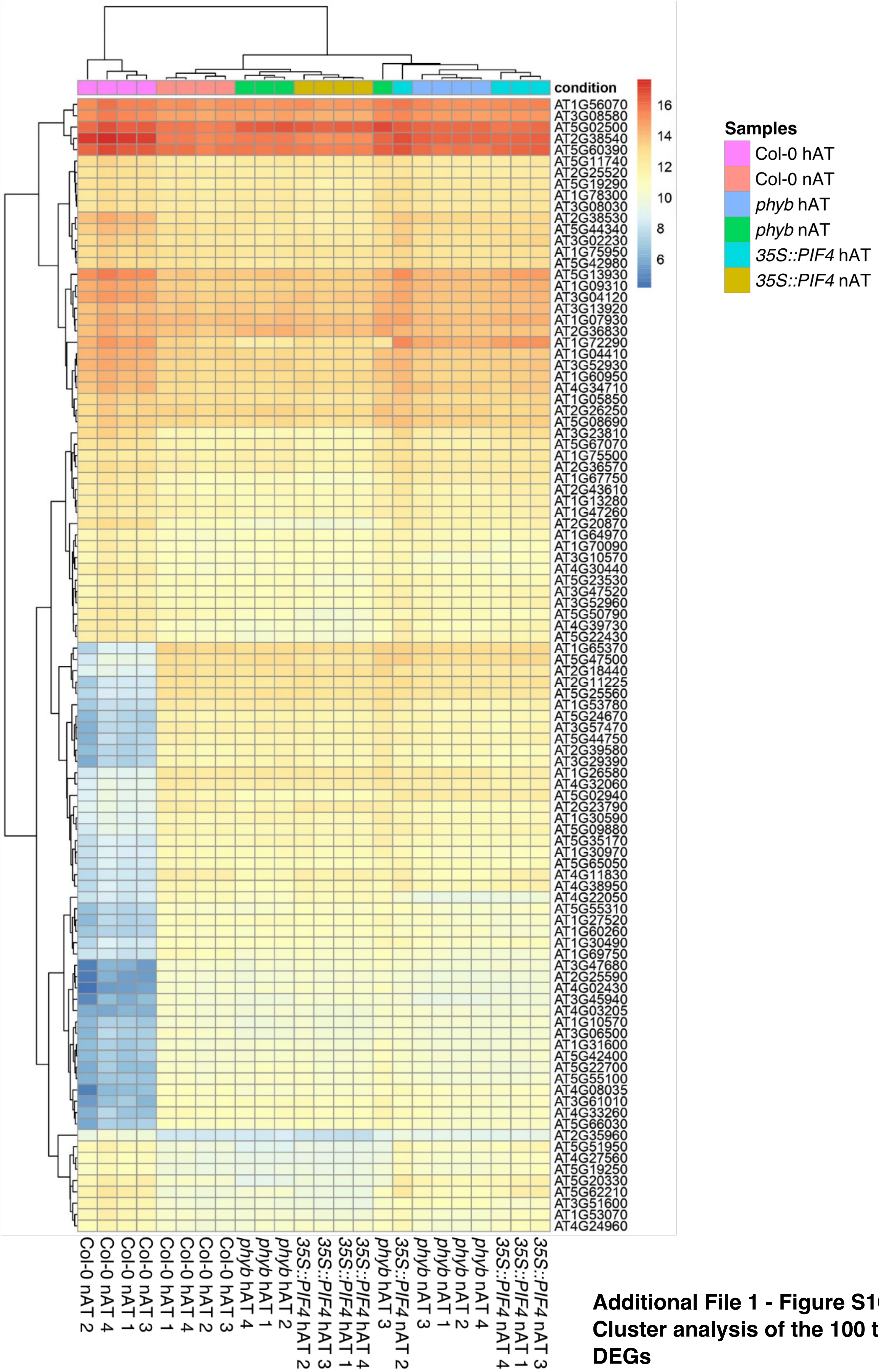

Additional File 1 - Figure S10. Cluster analysis of the 100 top DEGs
