# Supplementary material for: Integrative phenotyping analyses reveal the relevance of the phyB-PIF4 pathway in Arabidopsis thaliana reproductive organs at high ambient temperature
Source: BMC Plant Biol. 2024 Jul 29;24:721. doi: 10.1186/s12870-024-05394-w (PMC11285529; doi:10.1186/s12870-024-05394-w)
Supplement: Supplementary file 2 — Additional File 2 [file 12870_2024_5394_MOESM2_ESM.pdf]

Table S1 Quantification of lateral root density.

Table presents the mean number of lateral roots per centimeter of the primary root for each genotype/condition. This analysis includes data from 16 plants per genotype per condition in triplicate. Statistical differences were assessed by a two-way Analysis of Variance (ANOVA). Subsequently, *post-hoc* analysis using Tukey's test was conducted to identify specific group differences. Genotypes that share the same letter are not statistically different.

Genotype	Growth conditions	Averaged number of lateral root/cm of primary root	Statistical groups
Col-0	nAT	2.29	a
Col-0	hAT	1.61	b
35S::PIF4	nAT	1.77	b
35S::PIF4	hAT	1.59	b
phyB	nAT	1.54	b
phyB	hAT	1.01	c
pif4	nAT	2.12	a
pif4	hAT	1.47	b

Table S2 Mean of the length of the four longest lateral roots at maturity.

Table presents the mean of the length of the four longest lateral roots at maturity for each genotype/condition. The analysis encompasses 16 plants for each genotype Statistical differences were assessed by a two-way Analysis of Variance (ANOVA). Subsequently, *post-hoc* analysis using Tukey's test was conducted to identify specific group differences. Genotypes that share the same letter are not statistically different.

Genotype	Growth conditions	Averaged lateral root length (cm)	Statistical groups
Col-0	nAT	4.28	a
Col-0	hAT	9.20	b
35S::PIF4	nAT	5.16	a c
35S::PIF4	hAT	8.09	b
phyB	nAT	4.48	a c
phyB	hAT	6.09	c
pif4	nAT	4.26	a
pif4	hAT	8.53	b

Table S3. Statistical analysis related to Table 2. Anther abortion rate for different genotypes in nAT and hAT

Comparing anther phenotype for each genotype at hAT vs nAT		
**Genotype**	7 DAFD - *p*-values	9 DAFD *p*-values
Col-0	0.18242	0.02465 *
phyB	0.00491 *	0.00000 ***
35S::PIF4	0.00000 ***	0.00000 ***
pif4	1.00000	0.45918
pifq	1.00000	0.51969
* 0.05-0.001; ** < 0.001-0.0001; *** < 0.0001		
Comparing anther phenotype of each genotype with Col-0 at hAT		
**Genotype**	7 DAFD - *p*-values	9 DAFD *p*-values
phyB	0.39965	0.00005 ###
35S::PIF4	0.01527 #	0.00576 #
pif4	0.10287	0.08243
pifq	0.11841	0.03315 #
# 0.05-0.001; ## <0.001-0.0001; ### < 0.0001		
Comparing anther phenotype of each genotype at 7 with 9 DAFD under HT		
**Genotype**	*p*-values	
Col-0	0.275471698	
phyB	0.000436936^^	
35S::PIF4	0.540567636	
pif4	1	
pifq	0.532627646	
^ 0.05-0.001; ^^ <0.001-0.0001; ^^^ <0.0001		

The Fisher's exact test was performed for these comparisons. At least 40 anthers for each genotype/condition were examined. The experiments were performed in triplicate.

Table S4. Ovule defective phenotypes for the different genotypes at nAT and hAT

Genotype	Growth conditions	Normal	n	% of total defects	% collapsed embryo sac	% collapsed synergids	% unfused CC nuclei	% other defects
Col-0	nAT	122	129	5.4	0	5.4	0.0	0.0
Col-0	hAT	102	147	30.6	17.5	8.5	2.0	2.6
35S::PIF4	nAT	115	137	16.1	7.1	4.8	2.1	2.1
35S::PIF4	hAT	16	102	84.3	67.8	6.4	0.9	9.2
phyB	nAT	78	95	17.9	2.1	6.3	8.4	1.1
phyB	hAT	31	83	62.6	24.8	14.9	3.1	19.8
pif4	nAT	90	98	8.2	4.9	0.0	0.0	3.5
pif4	hAT	55	76	27.6	18.4	5.6	0.0	3.6
pifq	nAT	77	85	9.4	7.0	2.4	0.0	0.0
pifq	hAT	37	68	45.6	33.8	7.4	0.0	4.4

The experiments were performed in triplicate. Relates to Table 4. Ovule defective phenotypes for the different genotypes at nAT and hAT

Table S5. Transcriptomic data summary

Sample	replicate	clean reads	GC %	quality scores Q20	quality scores Q30
Col-0 nAT	1	42 060 406	46.01	98.07	94.13
Col-0 nAT	2	40 429 238	45.73	97.85	93.58
Col-0 nAT	3	40 740 204	45.72	97.96	93.84
Col-0 nAT	4	52 496 928	45.76	97.79	93.5
Col-0 hAT	1	52 031 214	44.63	98.05	94.04
Col-0 hAT	2	43 336 384	44.67	97.70	93.15
Col-0 hAT	3	47 869 856	44.70	98.04	94.14
phyB nAT	1	52 254 686	44.70	97.91	93.68
phyB nAT	2	48 593 332	45.14	97.97	93.80
phyB nAT	3	46 759 364	44.77	97.97	93.81
phyB nAT	4	52 854 984	44.69	98.00	93.96
phyB hAT	1	45 987 996	44.72	97.93	93.74
phyB hAT	2	45 703 752	44.83	97.93	93.95
phyB hAT	3	47 669 986	45.11	97.81	93.58
phyB hAT	4	65 471 960	45.03	97.81	93.58
35S::PIF4 nAT	1	43 886 772	45.13	98.34	94.99
35S::PIF4 nAT	2	50 746 592	44.72	98.02	93.95
35S::PIF4 nAT	3	65 893 110	44.99	98.06	94.21
35S::PIF4 nAT	4	55 254 998	44.83	98.15	94.29
35S::PIF4 hAT	1	46 380 078	44.91	97.97	93.85
35S::PIF4 hAT	2	43 825 406	44.64	98.03	93.98
35S::PIF4 hAT	3	48 510 456	44.67	97.88	93.60
35S::PIF4 hAT	4	42 629 628	44.57	98.01	93.91

Table S6. Mean of suspensor length for each genotype/condition.

Genotype	Growth conditions	Suspensor length (µm)	n	Statistical groups
Col-0	nAT	97.18	26	a
Col-0	hAT	86.14	23	c
35S::PIF4	nAT	110.96	29	b
35S::PIF4	hAT	74.86	5	c d
phyB	nAT	98.22	30	a
phyB	hAT	84.20	17	c
pif4	nAT	104.00	24	a b
pif4	hAT	75.66	8	c d
pifq	nAT	101.31	16	a b
pifq	hAT	66.90	27	d

The experiments were performed in triplicate. Relates to Figure 5. Statistical differences were assessed by a two-way Analysis of Variance (ANOVA). Subsequently, *post-hoc* analysis using Tukey's test was conducted to identify specific group differences. Genotypes that share the same letter are not statistically different.

Table S7. Seed size

The surface area of 1 000 seeds for each genotype were analyzed in triplicate at nAT and hAT. Relates to Fig. 9b. Statistical differences were assessed by a two-way Analysis of Variance (ANOVA). Subsequently, *post-hoc* analysis using Tukey's test was conducted to identify specific group differences. Genotypes that share the same letter are not statistically different.

Genotype	Growth conditions	Mean seed surface (mm^2)	Statistical groups
Col-0	nAT	0.0804	a
Col-0	hAT	0.1084	c
35S::PIF4	nAT	0.0814	a
35S::PIF4	hAT	0.1072	c
phyB	nAT	0.0847	b
phyB	hAT	0.1252	d
pif4	nAT	0.0837	b
pif4	hAT	0.1047	e
pifq	nAT	0.0790	a
pifq	hAT	0.1167	f

Table S8. Seed shape

Ratio of seed length over seed area of about 1 000 seeds for each genotype were analyzed in triplicate from plants grown at nAT and hAT. Relates to Fig. 9c. Statistical differences were assessed by a two-way Analysis of Variance (ANOVA). Subsequently, *post-hoc* analysis using Tukey's test was conducted to identify specific group differences. Genotypes that share the same letter are not statistically different.

Genotype	Growth conditions	seed length / area	Statistical groups
Col-0	nAT	5.1517	a c
Col-0	hAT	4.4843	d g
35S::PIF4	nAT	5.1986	a
35S::PIF4	hAT	4.3479	f
phyB	nAT	4.7850	b
phyB	hAT	4.1510	e
pif4	nAT	4.7850	b
pif4	hAT	4.5037	g
pifq	nAT	5.1286	c
pifq	hAT	4.3639	d f

Table S9. Number of seeds produced per silique.

The number of seeds per silique was calculated from at least 12 siliques per genotype from plants grown at nAT and hAT. Relates to Fig. 9d. Statistical differences were assessed by a two-way Analysis of Variance (ANOVA). Subsequently, *post-hoc* analysis using Tukey's test was conducted to identify specific group differences. Genotypes that share the same letter are not statistically different.

Genotype	Growth conditions	Averaged number of seeds per silique	Statistical groups
Col-0	nAT	62.16	a
Col-0	hAT	42.14	b
35S::PIF4	nAT	52.09	a
35S::PIF4	hAT	25.50	b
phyB	nAT	47.75	a
phyB	hAT	36.48	b
pif4	nAT	62.96	a
pif4	hAT	43.33	b
pifq	nAT	60.36	a
pifq	hAT	44.35	b

Table S10. The 100-seed weight.

The weight of 100 seeds from each genotype is measured in triplicate from plants grown at nAT and hAT. Relates to Fig. 9e. To assess differences per genotypes and between the two different conditions, a two-way Analysis of Variance (ANOVA) was performed. Subsequently, *post-hoc* analysis using Tukey's test was conducted to identify specific group differences. Genotypes that share the same letter are not statistically different.

Genotype	Growth conditions	100-seed mass (mg)	Statistical group
Col-0	nAT	1.80	a
Col-0	hAT	2.33	b d
35S::PIF4	nAT	1.93	a
35S::PIF4	hAT	2.30	b d
phyB	nAT	2.26	b d
phyB	hAT	3.25	c
pif4	nAT	1.93	a
pif4	hAT	2.26	b d
pifq	nAT	2.00	a b
pifq	hAT	2.56	d
